# The synapsin gene family in basal chordates: evolutionary perspectives in metazoans

**DOI:** 10.1186/1471-2148-10-32

**Published:** 2010-01-29

**Authors:** Simona Candiani, Luca Moronti, Roberta Pennati, Fiorenza De Bernardi, Fabio Benfenati, Mario Pestarino

**Affiliations:** 1 Department of Biology, University of Genoa, Viale Benedetto XV 5, 16132, Genova, Italy; 2 Department of Biology, University of Milan, Via Celoria 26, I-20133, Milan, Italy; 3 Department of Experimental Medicine (DIMES), Section of Human Physiology, University of Genoa, Italy; 4 Istituto Nazionale di Neuroscienze, Viale Benedetto XV 3, 16132, Genova, Italy; 5 Department of Neuroscience and Brain Technologies, The Italian Institute of Technology, Via Morego 30, 16163 Genova, Italy

## Abstract

**Background:**

Synapsins are neuronal phosphoproteins involved in several functions correlated with both neurotransmitter release and synaptogenesis. The comprehension of the basal role of the synapsin family is hampered in vertebrates by the existence of multiple synapsin genes. Therefore, studying homologous genes in basal chordates, devoid of genome duplication, could help to achieve a better understanding of the complex functions of these proteins.

**Results:**

In this study we report the cloning and characterization of the *Ciona intestinalis *and amphioxus *Branchiostoma floridae *synapsin transcripts and the definition of their gene structure using available *C. intestinalis *and *B. floridae *genomic sequences. We demonstrate the occurrence, in both model organisms, of a single member of the synapsin gene family. Full-length synapsin genes were identified in the recently sequenced genomes of phylogenetically diverse metazoans. Comparative genome analysis reveals extensive conservation of the SYN locus in several metazoans. Moreover, developmental expression studies underline that synapsin is a neuronal-specific marker in basal chordates and is expressed in several cell types of PNS and in many, if not all, CNS neurons.

**Conclusion:**

Our study demonstrates that synapsin genes are metazoan genes present in a single copy per genome, except for vertebrates. Moreover, we hypothesize that, during the evolution of synapsin proteins, new domains are added at different stages probably to cope up with the increased complexity in the nervous system organization. Finally, we demonstrate that protochordate synapsin is restricted to the post-mitotic phase of CNS development and thereby is a good marker of postmitotic neurons.

## Background

Synapsins are neuronal phosphoproteins that constitute a small family of synaptic molecules specifically associated with synaptic vesicles [1,2]. Synapsins regulate the balance between the readily-releasable pool and the reserve pool of synaptic vesicles and are involved in the neurotransmitter release and synaptic plasticity [3-7].

In vertebrates, synapsins are encoded by three distinct genes (*syn1-syn3*) that give rise to ten distinct alternative transcripts (*synIa/b, synIIa/b, synIIIa-f) *[8,9]. Invertebrate and vertebrate synapsin transcripts share similarity among three conserved domains known as A, C and E domains. In particular, the N-terminal region shows a stretch of homology in the A-domain containing the unique phosphorylation site for cAMP-dependent protein kinase (PKA) and calcium/calmodulin-dependent protein kinase I/IV (CaMK I/IV) common to all the synapsins, although the most extensive homology is found within the C-domain in a large region that represents the core of the synapsin transcript. A third conserved domain is located at the COOH-terminus (E domain) and it is shared by the a-type isoforms of synapsin I, II and III. In the last decades, a number of new functions have been proposed for synapsin family proteins, in addition to the classical role in neurotransmitter release. An involvement in maintaining vesicle integrity [10] and in regulating the proportion of functional vesicles [11-13] has been hypothesized.

Synapsins also modulate neuronal development such as establishment of neuronal polarity, neurite elongation and synapse formation [14,12-18]. In mammals, the three synapsin genes show a distinct temporal pattern of expression in neurons: synapsin III is expressed early during neuronal development and its expression is downregulated in mature neurons [16,19], whilst the product of the other two synapsin genes are upregulated at the onset of synaptogenesis and remain elevated in mature neurons [20].

Urochordates (i.e. ascidians and other tunicates) and cephalochordates (amphioxus) are chordate groups basal to vertebrates, with urochordates being the closest relatives to vertebrates [21]. Ascidian and amphioxus larvae posses simple central and peripheral nervous systems that reproduce well the basic organization of chordate nervous system and are therefore good models to investigate the molecular mechanisms underlying the chordate nervous system development [22,23]. In fact, in spite of the few neurons that constitute their nervous systems, a number of molecular studies have pointed out the expression, in both ascidian and amphioxus, of at least one member for virtually all the gene families identified in vertebrate development. Thus, synapsin genes, which are preferentially expressed in neurons, are useful markers for the investigation of neuron-specific gene expression.

In this study we have analyzed synapsin homologues in the ascidian *C. intestinalis *and amphioxus *B. floridae*, from a genomic and a developmental point of view. We demonstrate the occurrence, in both model organisms, of a single member of the synapsin family, and the presence of an alternative transcript exclusively in amphioxus. Furthermore, we have carried out a comprehensive comparative analysis of the exon-intron structure of the synapsin gene locus in humans and several metazoan phyla, and demonstrated a high level of conservation of its genomic organization. Such analysis was also extended to the proteins to reconstruct the domain evolution of synapsin along several metazoans. We conclude that protochordate synapsin is a neuronal-specific marker, which is expressed in several cell types of PNS (epidermal sensory neurons) and in many, if not all, CNS neurons during the final embryonic differentiation stages and in the fully developed embryonic nervous system.

## Results

### A single synapsin gene is present in basal chordates and in the sister-group of the bilaterian metazoans

**Figure 1:**
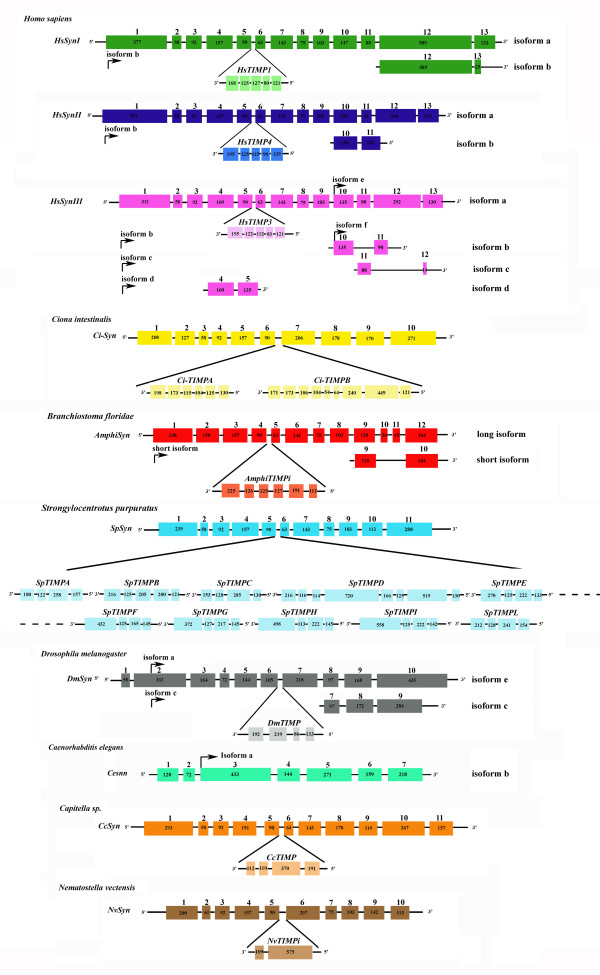
**Genomic organization of the synapsin locus in human and several invertebrate species**. The conservation of nested gene organization was really high when different species were compared. In all examined species, the TIMP gene is nested within the intron of Syn gene in reverse orientation, with the exception of *Caenorhabditis elegans*. *Ciona intestinalis *and *Strongylocentrotus purpuratus *are characterized by a peculiar nested organization of SYN-TIMP locus because more than one TIMP sequence was found (two TIMP in *C. intestinalis *and ten TIMP in *S. purpuratus*). Alternative transcripts generating by use of different exons are shown for *Homo sapiens *(isoforms a and b of *SYNI*, *II*; isoforms a-f of *SYNIII*), *Branchiostoma floridae *(long and short isoforms), *Drosophila melanogaster *(isoforms a, c and e), *C. elegans *(isoforms a and b). In particular, for the various transcripts we show with an arrow the start of the transcript, the different exons and only the last exon in common with the isoform having the longer ORF (isoform a for *H. sapiens*, isoform e for *D. melanogaster*, isoform b for *C. elegans *and long isoform for *B. floridae*). The base-pair length of each coding exon is indicated inside the boxes. The numbers above the colored boxes represent the exon numbering.

The amphioxus *B. floridae *and the ascidian *C. intestinalis *genomes contain a single synapsin gene (*AmphiSyn *and *Ci-Syn*) that shows a high degree of sequence conservation with vertebrate synapsins. The full length *Ci-Syn *cDNA was 1682 bp long and contained a protein-coding region of 1560 bp. The *Ci-Syn *cDNA translates into a polypeptide of 519 amino acids with a predicted molecular mass of 58 kDa. The exon/intron organization of the gene encoding *Ci-Syn *was deduced by comparing its cDNA sequence with the genomic sequences of scaffold 53 (release version 1.0) and chromosome 5 (release version 2.0) (Additional file 1). The exonic sequences in the genomic database and the corresponding regions of the transcript were 98% identical. *Ciona Ci-Syn *ORF was encoded in ~24 kb of genomic DNA in both scaffolds, and distributed in 10 exons with sizes between 58 and 271 bp (Figure 1).

In amphioxus, a single synapsin gene was identified by *in silico *analysis and PCR experiments. However, PCR assay on amphioxus embryonic cDNA library yielded two synapsin isoforms generated by alternative splicing of the same gene. These two cDNAs result in a long protein, termed AmphiSyn-long and a short truncated protein termed AmphiSyn-short, respectively of 474 and 447 amino acids. The *AmphiSyn-long *transcript consists of 12 exons (size between 36 and 248 bp) spanning 36 kb of genomic DNA of the scaffold 37 (Figure 1 and Additional file 1). The *AmphiSyn-short *transcript differs from the long form for the deletion of exon 10 and 11.

The ancestral condition of a single synapsin gene was previously demonstrated in some invertebrate species such as two ecdysozoans (the fly *Drosophila melanogaster *and the nematode *Caenorhabditis elegans*) [8,24] and three lophotrochozoans (the mollusks: *Loligo pealei*, *Aplysia californica *and *Helix pomatia*) [25-27]. Our results confirm that it was likely that a single synapsin gene was present in the chordate ancestor. Beyond analyzing the conservation at the chordates origin, we tested whether this conservation could be further extended in evolution. Systematic searches for synapsins-related sequences were carried out in the genome of a deuterostome (echinoderm *Strongylocentrotus purpuratus*), a lophotrochozoan (annelid *Capitella capitata*), and a diploblast, the cnidarian sea anemone *Nematostella vectensis*, representing one of the morphologically simplest metazoans. Such analysis confirmed that synapsin genes are metazoan genes present in a single copy per genome, except for vertebrates (Figure 1 and Additional file 1).

### Genomic organization of SYN locus

**Figure 2:**
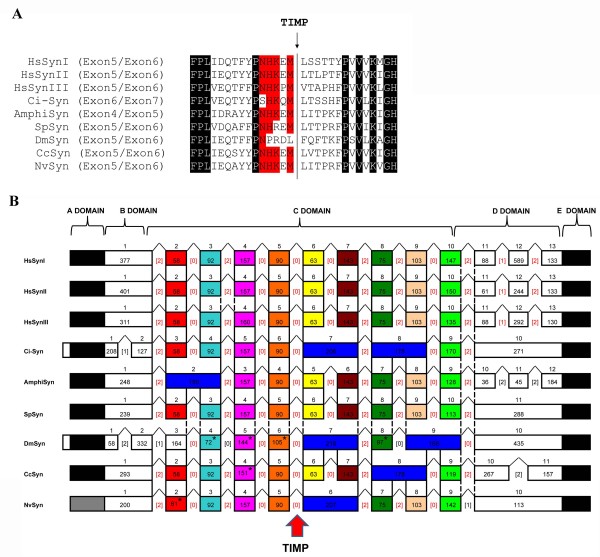
**Structure of the synapsin proteins domains and synapsin genes in representative metazoan phyla**. **A**: A multialignment of synapsin proteins corresponding to the bordering exons containing the introns encoding for TIMP sequences. Identical residues in all sequences are shown in black. Residues identical to the human sequences were indicated in red. **B**: Conservation of exon-to-protein domain correspondence. The contribution of individual exons or groups of two exons to the C-domain sequence is indicated by colored boxes. The black boxes indicate the contribution of first and last exons to the A- and E-domains. The white exons correspond to the highly divergent sequences of the B-domain and region between C and E domains. The grey box indicates that *Nematostella *lacks of a true comparable A domain. The orthologous exons found in all species are shown by the same color (an asterisk inside the box indicates when the length of exon differs from those of humans), whereas the blue boxes indicate when an invertebrate exon is split in two separate exons in humans. Solid lines and dotted lines represent identical intron locations and intron locations differing only for 5-18 bp, respectively. The base-pair length of each exon is indicated inside the boxes. The numbers above the colored boxes represent the exon numbering. Bracketed red numbers correspond to the common intron phase; the different intron phase is indicated by bracketed black numbers. Intron position encoding TIMP sequences is indicated by a red arrow. The diagram is not drawn to scale. See text for further details.

In mammals, each of the synapsin genes is associated with a single specific *TIMP *(tissue inhibitors of metalloproteinases) gene (Figure 1). The members of the TIMP family are specific endogenous polypeptide inhibitors of matrix metalloproteinases (MMPs), degrading enzymes that participate in the extracellular matrix turnover. In mammals, four *TIMP *(1-4) genes are known, three of which are nested with a specific *SYN *gene (*SYN1-TIMP1*, *SYN2-TIMP4*, *SYN3-TIMP3*), whereas a single *TIMP *(*TIMP2*) is independent of the SYN locus (Additional file 1). The nested TIMP proteins are similar in size and are encoded by five exons. In mammals, the gene structure of the SYN locus is highly conserved because the nested TIMPs are found at the same locations. To gain a more complete understanding of synapsin biology, we examined the evolutionary history of synapsin genes through genomic analysis of SYN locus of some protostome and deuterostome species and searched the genome sequences for member of TIMP families and identified independent TIMPs in some invertebrates (Figure 1 and Additional file 1). The nested SYN-TIMP organization appears to be maintained throughout metazoan evolution, with the only exception of the SYN locus of the *C. elegans *(Figure 1 and Additional file 1). A comparative analysis of the SYN-locus of several *Caenorhabditis *species together with that of the parasitic nematode *Brugia malayi *confirms that the absence of nested TIMP is a specific feature of the nematode's clade (Additional file 1). Furthermore, a multi-alignment of the synapsin proteins shows that the location of nested TIMP sequences is conserved in all analyzed species (Figure 2A) even if they are encoded by different introns. The SYN locus of amphioxus, *Capitella*, *Drosophila *and *Nematostella *is characterized by a single TIMP sequence within a single intron of the synapsin gene. Instead, the *Ciona *and *Strongylocentrotus *SYN loci possess two or multiple copies of TIMPs within one intron of the synapsin gene. Moreover, we identified independent and dependent TIMPs in both the amphioxus and *Nematostella *genomes, as well as in humans. However, due to the extreme divergence of both nested and independent TIMP sequences in invertebrates, it is not possible to identify their orthology with vertebrate TIMPs based on their amino acid sequences.

### Conservation of exon-to-protein domain correspondence

Next, we examined the synapsin gene structure (number of exons, gene length, orthologous exons, intron phases) to further define the evolution of the synapsin gene family (Figure 2B). All human synapsin genes exhibit similarities in their exon structure in both the NH_2_-terminal and central regions, whereas the COOH-terminal regions are more variable. In fact, the COOH-terminal region of the human paralogs is involved in alternative splicing of synapsin genes (Figure 1).

Comparison of the conservation of the exon/intron boundaries from representative metazoan phyla revealed the presence of several introns in conserved positions. Furthermore, several orthologous exons, in which the predicted amino acid sequences from invertebrates and humans can be aligned over the entire length (Figure 2B), were observed. We referred to the conservation of the protein domains (A-, C- and E-domains) that can be recognized in all species, although the relative position of B and D domains of human synapsin Ia were also considered.

The coding sequences of the C domain are conserved with respect to its exonic location, except for *Ciona *and *Drosophila *where the C domain begins with the third exon. The A domain is encoded by a sequence within the first exon; the only exception is found in *Ciona *and *Drosophila *synapsins in which the A domain begins respectively seven and thirteen amino acid residues from the NH_2_-terminus of the protein, and in *Nematostella *synapsin that does not have a clear counterpart of A domain in its first exon. The E domain is contained within the last exon in all sequences, although *Nematostella *has a highly divergent E domain.

The gene architecture of the C-domain is well conserved in terms of both intron phases and exon sizes in all analyzed metazoans (Figure 2B). However, some conserved intron loss events were observed. For instance, amphioxus has an exon 2 that is split in two exons in other species, whereas *Ciona*, *Drosophila *and *Nematostella *show a single exon (respectively 7, 7 and 6) corresponding to the mammal orthologous exons 6 and 7. Furthermore, *Ciona *has an intron loss at exon 8, that is also shown by *Capitella*. However, in general the C-domain intron loss events are well conserved across different invertebrate species.

Comparison of exon/intron organization at C domain level between human synapsin I and the invertebrate sequences shows that the organization of *S. purpuratus *is the most conserved (eight exons completely conserved in length), followed by the C-domain of amphioxus with six exons, *Nematostella *and *Capitella *with five exons completely conserved plus one orthologous exon that differs for very few nucleotides, and by *Ciona *with four completely conserved exons. The situation varies widely in *Drosophila *and *Caenorhabditis *that show highly divergent exonic sequences.

### Protein sequence and phylogenetic analysis

Vertebrate synapsins are a mosaic of multiple protein domains [28]. While the NH_2_-terminal portion contains two highly conserved domains named A and C, connected by the slightly less conserved domain B, the COOH-terminal portion arises from the combinatorial arrangement of numerous individual domains (domains D-J), including the highly conserved E domain which is shared among the a isoforms of the three human synapsin genes. Synapsins are excellent substrates for a variety of protein kinases. Protein phosphorylation sites have been extensively characterized in human synapsins. At least eight sites (sites 1-8) have been demonstrated experimentally in human synapsin I (Additional file 2) [3,29,30]. Sequence analysis allowed us to identify the three classical conserved domain A, C and E in the *Ciona *synapsin and in the two amphioxus isoforms. To gain a better understanding of the evolution of synapsin domains, we analyzed a multiple alignment of synapsin isoforms from *Homo sapiens *and several invertebrates (Additional file 3). Such analysis revealed that the highest degree of sequence conservation occurs in the C domain while a lower identity is found in E and A domains. However, there were no significant similarities with the other human domains.

Using pair-wise comparison, we found that both amphioxus and *Ciona *possess between 32-40%, 62-68%, 51-62%, identity to the A, C and E domains of humans, respectively (Additional file 4). At the same time, lophotrochozoans also share significant sequence identity in the three conserved domains. Interestingly, even if *Nematostella *seems to lack a true comparable A domain, and possesses a highly divergent E domain, it shares between 57-61% sequence identity with the C domain of humans, while *Drosophila *and *Caenorhabditis *display only 50% and 30% identity (Additional file 4).

The highly conserved C domain is the largest of the synapsin domains (approximately 300 amino acids). Studies on the crystal structure of the domain C suggest that it is structurally similar to ATP utilizing enzymes [31], with an ATP-binding domain. Such domain was detected in all analyzed synapsin proteins (Additional file 3). Moreover, all analyzed synapsins contain residues that are known to be responsible for ATP binding in mammals (Lys^225^, Lys^269 ^and Gly^276 ^in human synapsin I), except for *Caenorhabditis *synapsin in which there are Lys^225 ^Arg and Lys^269 ^Ser substitutions (Additional file 3).

The B-domain, as well as the domain connecting the C- and the E- domains, show little primary sequence identity between vertebrate synapsin I and invertebrate synapsins (Additional file 3). However, the proline-rich region between C- and E-domains is maintained in almost all invertebrate synapsins, although it is shorter than in human synapsins. The only exception was found in *Nematostella*, in which the linker region between C- and E-domains is too short to be considered as an effective proline-rich domain (Additional file 3).

The E-domain is a quite evolutionary-conserved region, especially for its COOH-terminal portion (~16 amino acids, see Additional file 3), supporting the finding that this region is of functional significance. In fact, the COOH-terminal region of E domain seems to be involved in modulating neurotransmitter release [25].

We also predicted the putative phosphorylation sites in the various invertebrate sequences by using PredPhospho and NetPhosK 1.0 and compared them with those of humans (Additional files 2 and 3). The most conserved consensus site is site 1 within A domain, which is a predicted site for phosphorylation by PKA and CaMKI/IV in all invertebrate synapsins except for *Nematostella*, which has a very divergent domain A (Additional files 2 and 3). The two phosphorylation sites for mitogen-activated protein kinase-extracellular signal-regulated kinase (MAPK/ERK) in the B-domain (sites 4, 5) are well conserved across invertebrate species, especially site 5, that is absent only in *Ciona *(Additional file 3). Interestingly, our prediction highlighted the presence of other putative phosphorylation sites in the C-domain (named A, B, C, D and E) and in E domain (named X and Y) (Additional files 2 and 3) that appear to be conserved in most of the analyzed species.

**Figure 3:**
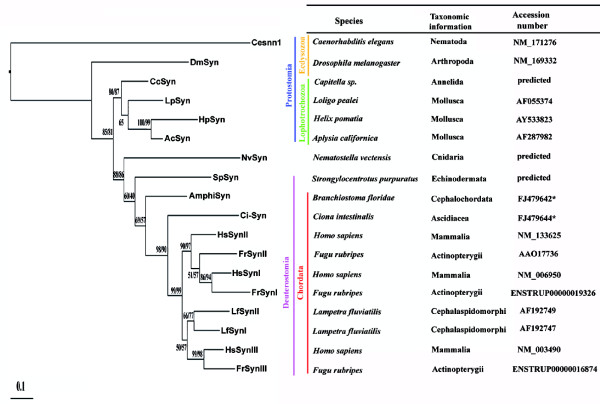
**Maximum likelihood phylogeny of synapsins from several vertebrate and invertebrate species**. The ML phylogeny of 18 representative synapsin proteins from various model organisms rooted at midpoint is shown. Numbers at nodes indicate the ML and NJ percentage bootstrap support with 1000 replicates (first and second values, respectively). Nodes supported only by the maximum likelihood analysis show a single bootstrap value. The scale bar indicates the number of amino acid substitutions per site.

To investigate the evolutionary history of synapsin family, we carried out phylogenetic analyses with sequences from representative species using both NJ and ML methods, yielding very similar results (Figure 3). In this study, we presented only the ML tree with bootstrap values from both NJ and ML analyses. Phylogenetic analysis using several invertebrate and vertebrate synapsin proteins locates Ci-Syn in proximity of the vertebrate clade synapsins, while AmphiSyn results shifted nearest the chordate common ancestor. Such results are highly supported (99/99 and 98/90 bootstrap support) and are consistent with recent phylogenies that place cephalochordates basal to {urochordates+vertebrates} in the chordate lineage. Moreover, *Nematostella *synapsin appears more closely related to the deuterostome synapsins as compared to that of *Drosophila *or *Caenorhabditis *ones. In addition, lophotrochozoans cluster together in a separate clade. Finally, based on sequence homology and phylogenetic analysis, amphioxus/*Ciona *synapsin could not be classified as being I-like, II-like, or III-like, as well as the lamprey synapsin I and II are not I-like o II-like but are basal to the synapsin III clade.

### *AmphiSyn *expression during amphioxus development

**Figure 4:**
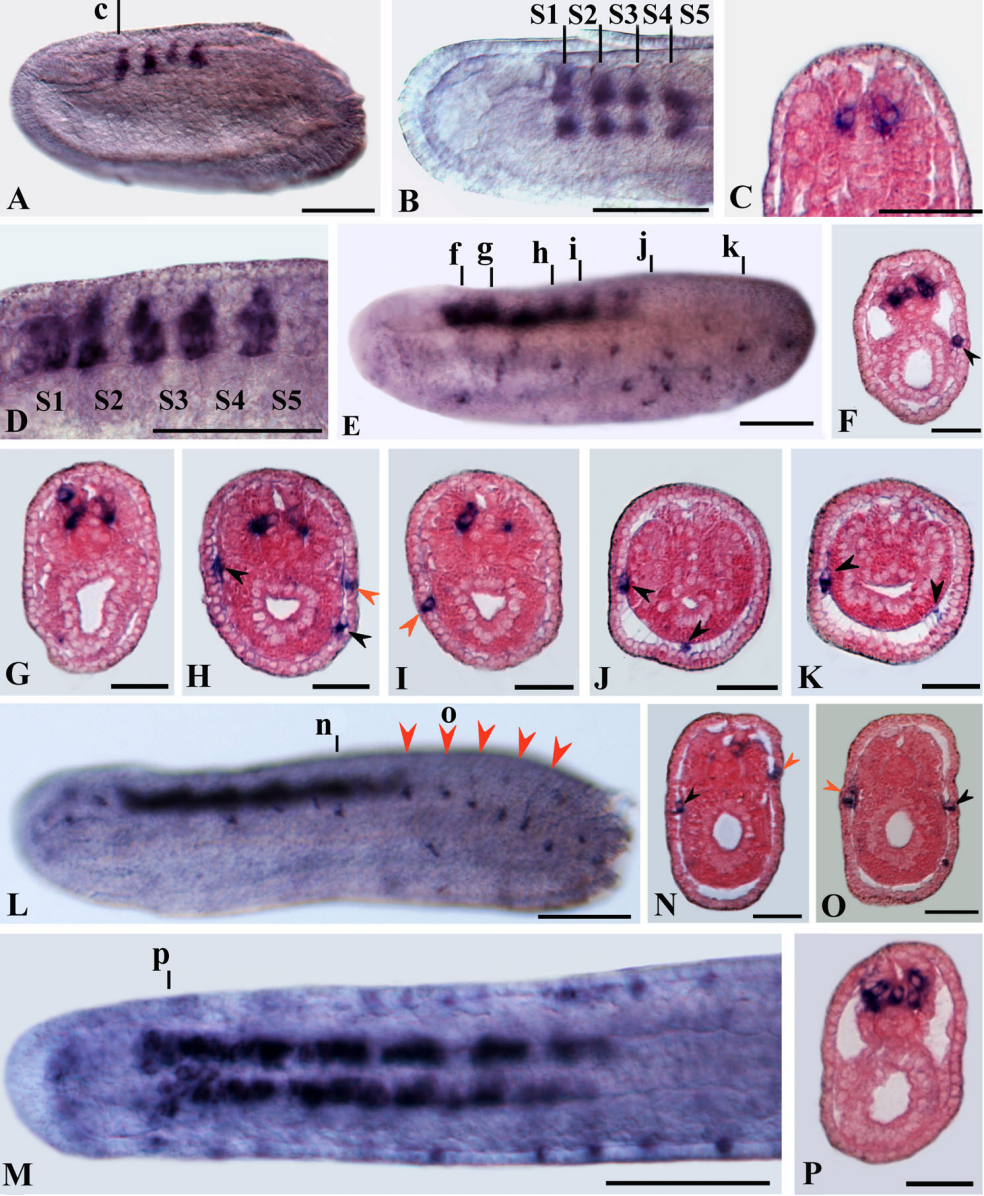
***AmphiSyn *expression in amphioxus neurulae**. Whole mounts in side views, except for B and M in dorsal view, anterior is to the left. Cross sections counterstained pink are viewed from the caudal end of the animal. Whole mount and cross section scale bars are 50 and 25 μm, respectively. A, B: 14-hour neurula showing expression in four spots of the neural plate at the somite boundaries, beginning at S1/2 (between somites 1 and 2) and extending to S4/5. C: Cross section through level c in A, showing a pair of ventrolateral labeled cells. D: Enlargement of 16-hour neurula showing a new cluster of labeled nerve cells at level of the posterior part of somite 1. E: In 18-hour neurula *AmphiSyn *expression extends in the posterior neural tube and a new expression appears in single epidermal cells in the ventro-lateral region of the embryo. F-K: Cross sections trough levels shown in E. The labeled sensory cells are mostly confined into the subepidermic layer (black arrowheads). Few positive sensory cells were located in epidermal layer (red arrowheads). In the nerve cord, labeled cells were mainly located ventrolaterally (F-I), except for few cells located dorsolaterally (E, G). L: In 20-hour neurula the most caudal epidermal labeled cells showed a periodically arrangement following the somite boundaries (red arrowheads in L). M: Dorsal view of two-thirds of the specimen in L. N-P: Cross sections through levels shown in L and M.

The expression of *AmphiSyn *was examined during amphioxus embryonic and larval development by whole mount *in situ *hybridization. Transcripts were first detected in 14-hour neurulae in four cell clusters of the neural plate (Figures 4A-C). Such clusters are periodically arranged following the boundary of the somites, beginning at S1/2 and extending to S4/5. Closer examination of such embryos in cross section (Figure 4C) revealed that transcripts were primarily confined on either side of the ventral midline. The same periodicity was also maintained in 16-hour neurulae even if, in front of S1/S2 boundary, a further rostral cluster of cells appeared (Figure 4D). As development proceeded, *AmphiSyn *expression extended in the posterior neural tube, but the periodicity of positive cell clusters became less evident (Figure 4E). Cross sections localized the expressing cells throughout the ventral extent of the neural tube (Figures 4F-I) except for few positive dorsal nerve cells (Figure 4G) that increased just after the first dorsal ocellus (Figure 4E). By 18 hr, a new expressing domain became evident in single epidermal cells located ventro-laterally along the body of the embryo. Such epidermal cells were preferentially localized in the sub-epidermal layer (Figures 4F, H, J, K), although few labeled cells also appeared to reside in the epidermal sheet (Figures 4H, I). Two distinct cell populations in the sub-epidermal sheet were found: the first one was characterized by spherical cells preferentially localized in the mid-ventral region (Figure 4J); the second consisted of elongated cells residing in the ventro-lateral region (Figures 4H, J, K). At the late-neurula stage (20 hr), the expression in epidermal cells shifted in a more dorsal region (Figure 4L). At this stage, we observed a synchronized development between the epidermal cells expressing *AmphiSyn *and the underlying somites (Figure 4L). In the neural tube, transcripts starting at level of the posterior cerebral vesicle, remained through the two thirds of the total length of the embryo (Figures 4L, M). The initial clusters of nerve cells observed in mid-neurulae had now expanded into clear longitudinal rows (Figure 4M). However, the most anterior domain of *AmphiSyn *expression was characterized by a cluster of ventro-lateral nerve cells (Figure 4M).

**Figure 5:**
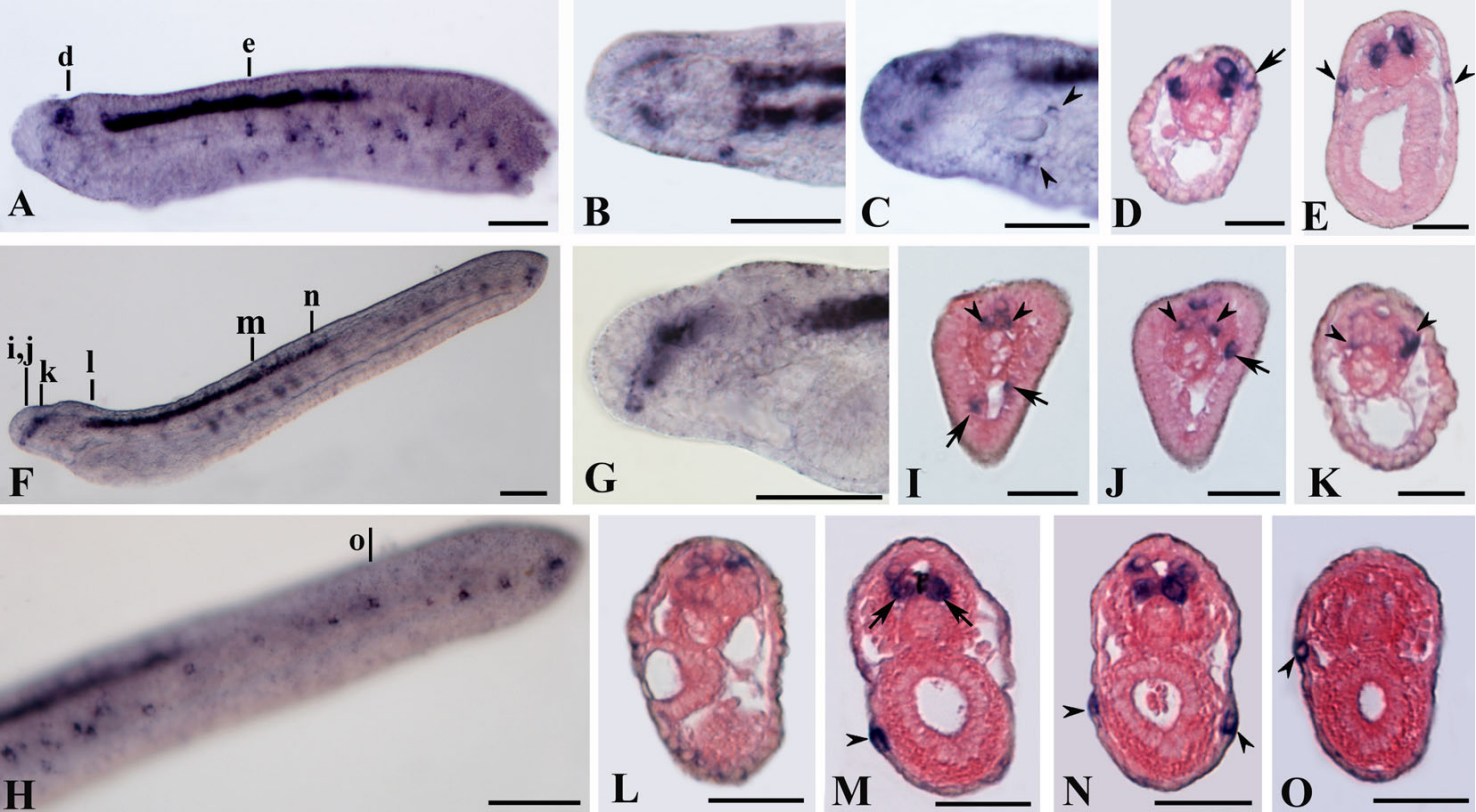
***AmphiSyn *expression in amphioxus larvae**. All embryos are oriented with the anterior side to the left. Cross sections are viewed from the caudal end of the animal. Whole mount and cross section scale bars are 50 and 25 μm, respectively. A: Side view of 24-hour larva. *AmphiSyn *expression was found in the most rostral region of the cerebral vesicle. Expression also occurs in the two-third of the nerve cord. B, C: Enlargement of the rostral region of specimen in A in dorsal and lateral views. The latter shows clearly two large epidermal cell bodies localized around the preoral organ. D, E: Cross sections trough levels shown in A. In the rostrum, some positive epidermal cells near the blastopore were visible (D) (arrow). All sensory cells at this stage were localized only in the epidermal sheet (black arrowheads). F: 36-hour larva in lateral view. Sensory cells were visible in the rostrum and in the lateral body. A high concentration of labeled sensory cells was found in the middle of the body. G: High power view of the head of the specimen in F, showing *AmphiSyn *expression in rostral cells located more ventrally. I-K: Cross sections through levels shown in F. *AmphiSyn*-positive photoreceptor cells of frontal eye complex (arrowheads) were visible. Presumptive cells located under the epidermis surface in the ventral portion of the rostrum were visible in I and J (arrows). Such cells could correspond to the precursors of corpuscles of de Quatrefages. H: Enlargement of the most caudal region of the specimen in F with in focus epidermal sensory cells located mid-laterally along the flanks of the body. L-O: Cross sections through levels shown in F. In M, photoreceptor cells of dorsal ocellus express *AmphiSyn *(arrows).

By early larval stage (24 hr), a new expression domain appeared in the most anterior tip of the cerebral vesicle (Figures 5A-C), in a region where the frontal eye complex will develop. Scattered epidermal cells along the flanks of the body were still present (Figures 5A and 5E) and several cells were just visible around the preoral organ (Figure 5C) and the rostrum (Figures 5A, C, D). By this stage, all sensory labeled cells were found exclusively in the epidermal sheet (Figure 5E). At 36-hour larva, the expression in the neural tube remained substantially similar to that of the preceding specimen, although some further dorsal cells appeared to express *AmphiSyn*. The latter were more abundant in the nerve cord just behind the first dorsal Hesse ocellus (Figures 5F, M, N), but very few positive cells were also found in the presumptive region of lamellar organ (Figure 5L). Moreover, photoreceptor cells of dorsal Hesse ocellus (Figure 5M) as well as that of the frontal eye complex were clearly stained (Figures 5G-K). *AmphiSyn *expression was never found in the most posterior part of the embryonic and larval nerve cord, probably because of the delayed differentiation of neurons in the more caudal regions. By 36 hours, several epidermal cells along the midline of the flanks continued to express *AmphiSyn *(Figures 5F, H, M, N, O), as well as further epidermal cells located in the rostrum in a ventro-lateral position (Figure 5G). The latter cells were clearly located in the subepidermal connective tissue (Figures 5I and 5J) and probably correspond to the sensory cells precursors of the corpuscles of de Quatrefages.

### *Ci-Syn *expression during *Ciona *development

**Figure 6:**
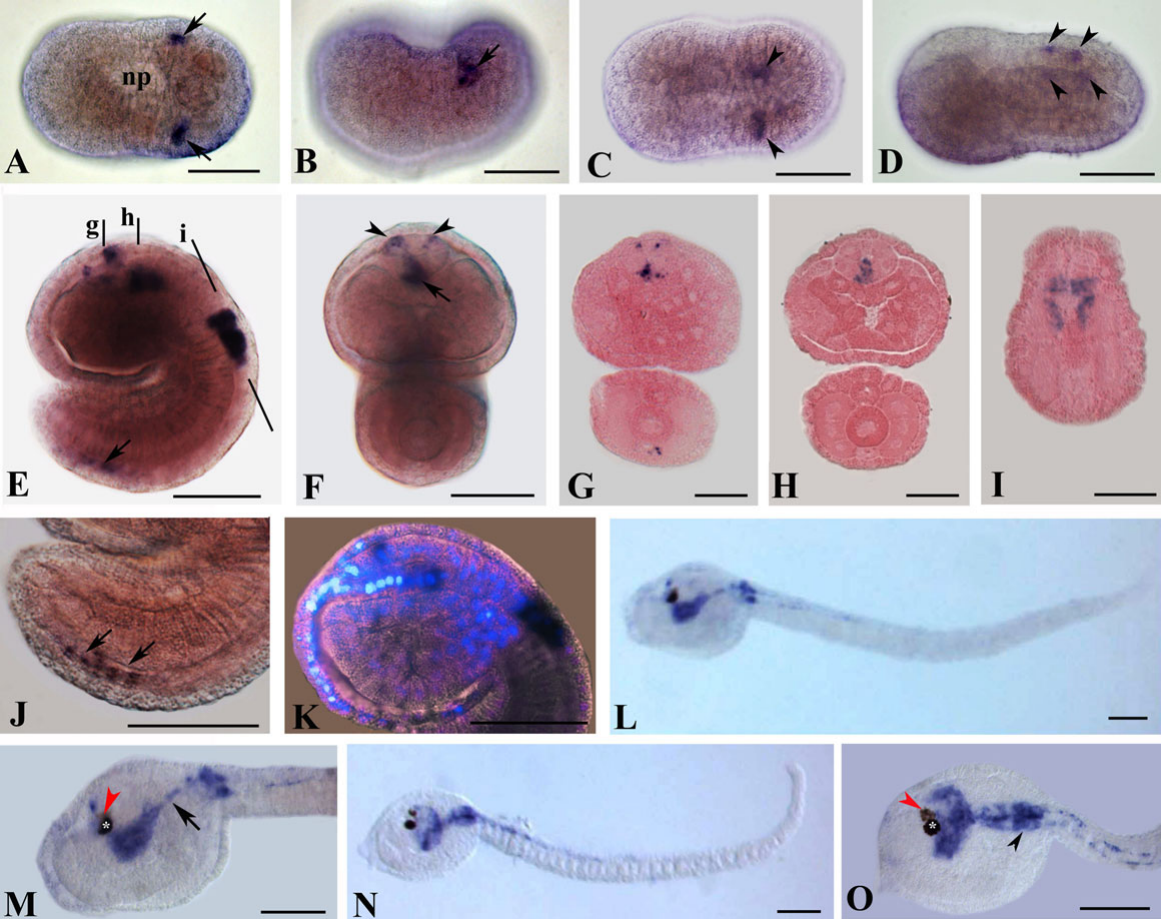
***Ci-Syn *expression at the neurula and tailbud stages**. Whole mounts in side view, except for A, C in dorsal view, anterior is to the left. Cross sections viewed from the caudal end of the animal. Scale bars = 50 μm in whole mounts; 20 μm in cross sections. A, B: Mid-neurula stage embryos in dorsal (A) and lateral (B) view. *Ci-Syn *signal is present in two lateral spots (arrows) located posterior to the neuropore (np). C: Dorsal view of a late neurula embryo. The two *Ci-Syn *positive spots (arrowheads) are closer to the midline than in the previous stage. D: Lateral view of an initial tailbud embryo. Four bilateral symmetrical spots (arrowheads) are aligned along the posterior dorsal midline. E-K: Mid-tailbud embryos. E: Lateral view. *Ci-Syn *expression is found in the sensory vesicle, in the visceral ganglion and in some cells near the posterior end (arrow). In front view embryo (F) and in cross sections through levels shown in E (G, H), it appears that, in the sensory vesicle, positive cells are aligned along the posterior floor (arrow) and are present in the anterior roof in bilateral symmetrical spots (arrowheads). I: Cross section at the level of the visceral ganglion as indicated in E. J: Enlarged view of E showing the positive dorsal neurons near the tip of the tail. K: Superimposition of *in situ *hybridization with DAPI staining. Four nuclei are visible in correspondence of *Ci-Syn *signal in the posterior sensory vesicle and eight nuclei are present in the visceral ganglion. A single nucleus is visible in correspondence of the dorsal signal in anterior dorsal sensory vesicle. L-O: Prehatching larvae. M, O: *Ci-Syn *transcripts are present in posterior sensory vesicle, in fibers connecting the sensory vesicle to the visceral ganglion (arrow), in cells of the visceral ganglion (arrowhead). white asterisk, pigment cell of otolith; red arrowheads, pigment cell of ocellus.

Spatial expression pattern of *Ci-Syn *was examined in embryos and larvae by whole-mount *in situ *hybridization. *Ci-Syn *expression was first detected at the mid neurula stage (E39, 6.8 hpf, hatching post fertilization), in two lateral symmetrical cells at the posterior end of the neural folds (Figures 6A and 6B). At late neurula stage (E42, 7.4 hpf), when the neural tube closure had begun in the posterior territories, the two *Ci-Syn *positive cells moved closer to the dorsal midline (Figure 6C). In initial tailbud embryos (E48, 8.45 hpf), two additional spots of hybridization signal appeared anterior to the ones already present in earlier stages (Figure 6D). In mid tailbud embryos (E70, 10 hpf), when the neuropore was closed and the neurulation was completed, *Ci-Syn *expression also appeared in cells of prospective anterior nervous system. In particular, *Ci-Syn *transcripts were present in the dorsal anterior sensory vesicle in two bilateral symmetrical cells, and in two more small rostral cells, near the opening of the neuropore (Figures 6E and 6F). From cross sections of hybridized embryos (Figures 6E and 6F) and by staining nuclei with DAPI (Figure 6K), it was possible to distinguish four positive cells aligned in a single row in the posterior sensory vesicle floor plate and four pairs of positive cells in the region that will contribute to form the visceral ganglion (Figures 6I, K). Further few positive cells were localized at the caudal tip of the neural tube (Figure 6J). In late tailbud embryos (E77/13.5 hpf, E91/15.9 hpf), *Ci-Syn *transcripts were broadly present in the posterior sensory vesicle and in the visceral ganglion (Figures 6L-O). Moreover, *Ci-Syn *transcripts were detected in fibers connecting the sensory vesicle to the dorsal side of the visceral ganglion and in fibers emerging from the ventral side of the visceral ganglion and running into the tail (Figure 6M, O). These last positive fibers extended for the first third of the length of the tail (Figure 6L, N). *Ci-Syn *expression was also detected in cells located in the anterior region of the tail, recently described by Horie et al. [32] and named anterior caudal inhibitory neurons (ACIN).

**Figure 7:**
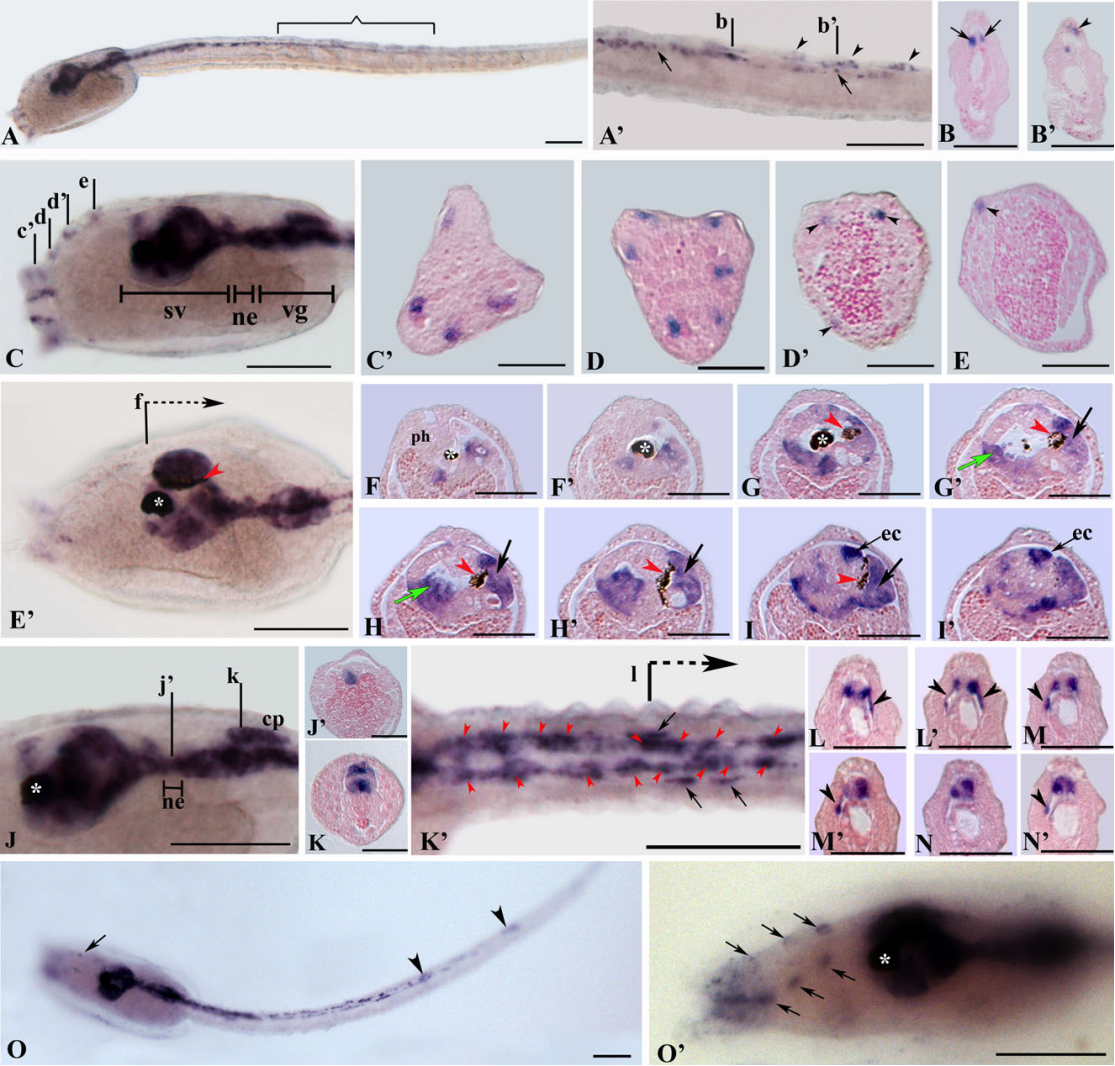
***Ci-Syn *expression at the larval stage**. Whole mounts in side view, except for E', J, O in dorsal view, anterior is left. Scale bars = 50 μm in whole mounts; 20 μm in cross sections. A-N': Newly hatched larva. A: Side view, the signal extends from the sv until two/third of nerve cord (nc). A': Enlargement of the tail. *Ci-Syn *is expressed in three bipolar neurons (arrowheads). Small spots are also visible along the nc (arrows). B, B': Cross sections through levels in A' showing the dorsolateral fibers (arrows) and a bipolar neuron (arrowhead). Moreover, a small positive dot is visible. C: Lateral view. Expression is found in sv, ne, vg, neurons of adhesive papillae and some RTEN. C'-D: Cross sections through levels in C showing the expression in papillar neurons. D', E: Cross sections through levels in C. *Ci-Syn *expression occurs in some RTEN (arrowheads). E': Dorsal view of the specimen in A showing expression in several regions of the sv. Expression is also detected in the ne and the vg. F-I': Cross sections through levels in E'. J: Enlargement of specimen in C in side view. J', K: Cross sections through levels in J. J': Transcripts are in the ventral neck region. K: A signal is found in ventral motor neurons and in dorsal cp. K': Enlarged dorsal view of the tail. Red arrowheads indicate anterior caudal inhibitory neurons (ACINs); arrows indicate motor neuron endplates. L-N': Cross sections through levels in K'. *Ci-Syn *is expressed in ACINs and in ventrolateral elements (arrowheads). O-O': Late larva. O: Dorsal view, the arrow indicates one RTEN; arrowheads indicate bipolar neurons. O': Enlargement of the trunk showing positive RTEN (arrows). cp, contrapelo cells; coronet cells (green arrow), ec, eminens cell; ne, neck; ph, pharynx; pigment cell of ocellus (red arrowhead in E'and G-I); sv, sensory vesicle; vg, visceral ganglion; white asterisk, pigment cell of otolith.

In the hatching larva, *Ci-Syn *transcripts were present in the peripheral and central nervous systems. In the tail, *Ci-Syn *expression had extended to the anterior two thirds of length (Figures 7A and 7A'). Histological cross sections showed that *Ci-Syn *transcripts were present in axons of lateral nerves and in some motor neuron endplates. A strong hybridization signal was present in the sensory vesicle and in the visceral ganglion (Figures 7A, C, E). Histological cross sections showed that *Ci-Syn *transcripts were asymmetrically distributed and localized in all major neuron clusters: the photoreceptors cells, the pressure receptor coronet cells, the big dorsal eminens cell. In the neck, *Ci-Syn *expression was detected in the ventral region, while in the visceral ganglion the gene was widely expressed in both dorsal and ventral regions, where respectively contrapelo neurons and motor neurons are localized. Expression in ACINs, in their endplates expanding laterally to the muscular mass, and lateral running fibers was still present (Figure 7J). These neurons, formerly named planate neurons for their flat ovoidal shape [33], were at least eight and their somata were located within the nerve cord, as it was clearly visible in cross sections (Figures 7J'-N). Gene expression was also detected in some cells of the peripheral nervous system, and in particular in the sensory neurons of the palps and in some of the rostral trunk epidermal neurons (RTEN) described by Takamura [34]. In the tail, at least two, often three, large dorsal epidermal neurons were labeled. Such cells correspond to the bipolar GABAergic interneurons [35]. After hatching, the nervous system of the larva is still developing. Accordingly, the expression pattern of *Ci-Syn *extended more in a posterior position in the tail in late larva than in the hatching one and more numerous RTEN appeared positive in anterior dorsal position (Figures 7O-O').

## Discussion

### Antiquity of SYN locus genomic organization

In this study, we have analyzed the molecular evolution of the synapsin gene family. Our analysis revealed that protochordates, as well as several invertebrates, possess a single synapsin gene, whereas higher vertebrates have three synapsins genes. It is, therefore, safe to assume that prior to the two rounds of vertebrate genome duplication (2R hypothesis) [36], only a single synapsin gene existed. On the other hand, at least two synapsin genes have been identified in the lamprey and three genes are present in higher vertebrates [8]. Moreover, synapsin genes from lower organisms appeared to have a genomic architecture similar to the vertebrate synapsins. Such conservation has mainly occurred in the C-domain, although some conserved introns loss events were observed.

A comparison of the synapsin locus between mammals and several invertebrate species showed that the nested organization of TIMP and synapsin is a general feature that has been conserved during evolution. A well-conserved guest/host relationship is that of the TIMPs within the synapsin gene family (*Syn1/TIMP-1*, *Syn3/TIMP-3 *and *Syn2/TIMP4*), which is maintained from *Drosophila *to human [37]. Here we demonstrate that such genomic organization arose very early during metazoan evolution, being also present in the sea anemone, *N. vectensis*. These data match the new theory that before the emergence of bilateral animals, cnidarians split from the main animal lineage and their genome retained many of the features present in the last common ancestor of animals.

In addition, our results demonstrate that the ancestral Syn-TIMP locus has undergone different duplication events. In fact, TIMP not nested within a synapsin gene, previously demonstrated only in vertebrates, was also found in *Nematostella *and amphioxus. Interestingly, two deuterostomes not having an independent TIMP, such as sea urchin and *Ciona*, possess a peculiar TIMP organization with more than one TIMP within the intron of the synapsin gene. Such data support the view that the ancestral organization of SYN locus resembles much more that of amphioxus and *Nematostella*, whereas the genomic organization of urochordates and echinoderms was lineage-specific and more divergent. We conclude that strong stabilizing pressures have been enforced throughout metazoan evolution to maintain the comparable genomic organization of present-day synapsin gene locus across diverse taxa.

### Evolutionary conserved domain architecture of synapsin proteins

In the present work, we show that almost all invertebrate synapsins share similarities among the evolutionary conserved domains A, C and E, even if *Nematostella *lacks a clear A-domain. Therefore, the multiple synapsin domains seem to have evolved at different rates throughout evolution. During the evolution of vertebrates, at least two gene duplication events can be hypothesized to give rise to the synapsin gene family and these events were probably accompanied by the emergence of additional domains (D-J).

In order to obtain a more exhaustive characterization of the evolution of synapsin domain architecture and their relationships in metazoans, we took advantage of the wide range of genomes now available (see Additional file 5). BLAST searching in the current genomic and EST databases of other basal, *i.e*. diploblastic, animals failed to identify synapsin-related sequences in sponges (*Amphimedon queenslandica*), while positive results were obtained from other cnidarians species including *Hydra magnipapillata *and *Acropora millepora*. Unfortunately, these sequences are clearly incomplete, due to gap in the genome and EST and/or difficulties to predict complete open reading frame. In addition, no synapsin sequences have been identified in the *Trichoplax adhaerens*, representing the basal metazoan phylum *Placozoa*, as well as the choanoflagellate *Monosiga brevicollis*, which branches as the closest known outgroup to metazoans [38]. Outside metazoans, synapsin-related sequences were retrieved from protozoans: in the amoeba-flagellate *Naegleria gruberi *and in the amoebozoa *Entamoeba histolytica *(Additional file 5). Interestingly, the sequences identified in these two protozoans encode for two proteins characterized by a single domain sharing significant sequence identity with the C-domain of invertebrates and vertebrates synapsins (Additional file 6).

**Figure 8:**
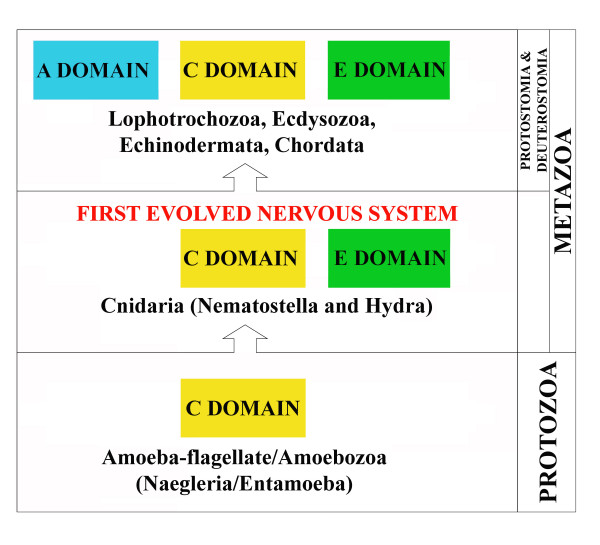
**Schematic diagram showing the possible evolution of synapsin protein domains**. The colored boxes highlight the insertion of new conserved domains at different evolutionary nodes shown on the right. See the text for further details.

Thus, one possible hypothesis on the evolution of synapsin proteins is that new domains were added at various stages of evolution probably to cope up with the increased complexity in protein functions which paralleled the increased complexity of the nervous system (Figure 8). In particular, we suggest that: 1) a single C-domain was already present in protozoans; 2) the E-domain appeared to have co-evolved with C-domain in basal metazoans. The conservation of the E-domain over such a wide phylogenetic distance, suggests that it shares a function common to all eumetazoans (cnidarians + bilaterians); 3) three conserved domains (A, C and E) appeared with protostomia and subsequently were maintained in deuterostomia and chordates (Figure 8). Thus, the emergence of E domain appears to be correlated with the beginning of the genesis of nervous organization, since the simplest metazoans known to possess a nervous system are those of the phylum *Cnidaria *[39].

### Alternative transcripts in the synapsin gene family

Mammal synapsins comprise a family of at least ten members (*SYN Ia, Ib, IIa, IIb, IIIa-IIIf*) generated by alternative splicing of three genes. In human, the genes for *SYNI, SYNII, SYNIII *contain 13 exons (Figure 1). The last exon of *SYNI *gene contains two splice acceptor sites whose alternative use generates the two isoforms *SYNIa *and *Ib *encoding for proteins of 705 and 669 amino acids, respectively. The two isoforms of *SYNII *gene (*IIa *and *IIb*) originate from alternative splicing involving the last three exons. Finally, *SYNIII *gene gives rise to several isoforms (*IIIa-IIIf*). The isoforms b and c are identical to *SYNIIIa *except for the absence of exon 11 (in *IIIb*) and exon 12 (in *IIIc*) and the difference in the reading frames of the last exon (Figure 1). The other isoforms are very peculiar being the result of two different alternative splicing events involving two new exons between exons 5 and 6 (in *IIId*) and exons 9 and 10 (in *IIIe, f*) (Figure 1) [9].

In invertebrates, Kao and coworkers [8] described the synapsin gene of *C. elegans *as composed of 5 exons, but suggested the existence of one extra exon upstream of exon 1. By using genomic and EST sequences we can now state that *C. elegans *has a single *Syn *gene, composed of 7 exons, giving rise to two different transcripts, *Cesnn1a *and *Cesnn1b *(Figure 1). The *Cesnn1b *is identical to the *Cesnn1a *except for a sequence at the 5' end of the coding region found only in the isoform b and originated by the use of two exons at the most 5' end.

Alternative transcripts have been also described in *Drosophila*, *Aplysia californica*, *Loligo pealei *[24-26]. In *Drosophila*, the single synapsin gene leads to 5 different isoforms. Three of them (isoforms a, c and e) (Figure 1) differ at the COOH end of the molecule (Additional file 3). Moreover, the isoforms a and c lack a sequence at the most 5' end. In *Drosophila*, two large synapsin isoforms (d and f) generated by UAG read-through were also found. In *Aplysia *four alternative transcripts derived from a single synapsin gene has been described. Such splice variants (called 11.1, 2.1, 7.1 and 8.2) differ for two inserted sequences and one possible substitution located in the most NH_2_-terminus and in the central part of the molecule (Additional file 3). Such isoforms are more similar to those of invertebrates than to those of vertebrates. In *Loligo pealei*, two transcript variants (*s-syn-short *and *s-syn-long*) differing only by a 111-bp deletion/insertion, located in the COOH-terminal half, are present (Additional file 3). In amphioxus, we demonstrated the occurrence of a short and a long isoforms in the comparable region of squid synapsin (Additional file 3). The amphioxus short isoform differs from the long form for the deletion of exons 10 and 11 (Figure 1).

Looking at the splicing events impacting known functional domains of the synapsins, we can suggest that invertebrate isoforms are not comparable to those of the vertebrates (Additional file 3). In fact, the major splicing events in vertebrate synapsin transcripts encode for truncated proteins, missing the COOH-terminal end of the E-domain. In contrast, some invertebrate alternative splice forms (such as those of *Drosophila *and *Caenorhabditis*), involving the NH_2_-terminal coding region, affect the A-domain, whereas others (such as those of amphioxus and squid) alter the domain that follows C domain. Only the isoform c of *Drosophila*, lacking the last part of E domain, shares some similarity with the alternative splice form Ib of human synapsin (Additional file 3). Finally, the highly sequence identity within the E-domains of vertebrate synapsins Ia, IIa, IIIa, and the evolutionary conservation of such domain provide evidence that the ancestral synapsin gene encoded for an a-like synapsin isoform.

### Synapsin expression in post-mitotic phase of CNS development in amphioxus and *Ciona*

Early during development, some organisms, such as nematodes and ascidians, show a remarkably invariant pattern of cell divisions [40-42], and the pattern is so stereotyped that each cell can be individually named. Such type of developmental process has been extensively used to precise embryonic origins of the various neuronal types in these model organisms.

Since ascidian embryos show such invariant pattern of early cleavage, it has been possible to trace a mitotic descendant map of cells forming the CNS [42]. Interestingly in *Ciona*, all cells that express *Ci-Syn *had ceased their mitotic activity. Cells within the prospective sensory vesicle cease division as 13^th^-generation, with the exception of four cells that become post-mitotic in their 10^th ^(1), 11^th ^(2), or 12^th ^(1) generation. The position of the two *Ci-Syn *positive cells in mid-neurula embryos is compatible with that of the A/A10.57 cells, which had ceased mitotic activity in their 10^th ^generation. In initial tailbud embryos, the observed positive spots most probably correspond to the A/A11.117 and A/A11.118 cells, daughters of A/A10.59, which had entered the post-mitotic phase in their 11^th ^generation. Up to this stage, *Ci-Syn *transcripts are restricted in cells of the "A" line. By mid tailbud stage, after neurulation is completed, other cells become post-mitotic, including cells of the "a" line of prospective central nervous system, and in parallel *Ci-Syn *expression extends to these territories. In fact, in mid tailbud embryos *Ci-Syn *transcripts are present in the dorsal anterior sensory vesicle in the precursors of otolith and ocellus (a/a 10.97) and in two more rostral small cells, near the opening of the neuropore, most probably a/a 10.102, that will contribute to form the neurohypophyseal complex of the larva. Moreover, at this stage *Ci-Syn *is expressed in a row of cells in the floor plate of the posterior sensory vesicle, corresponding to A/A 10.27 and A/A 10.28 post mitotic cells. By this stage, an additional pair of cells becomes post-mitotic in the prospective visceral ganglion: the A/A 12.239 cells. Together with the A/A 10.57, the A/A 11.117 and the A/A 11.118, that had ceased mitotic activity in earlier stages, form a group of eight paired neurons located in the ventro-lateral position of the visceral ganglion, where cholinergic motor neurons have been identified in hatching larva [43]. Thus, cholinergic neurons are the first ones to differentiate in the embryos. Other post mitotic cells derive from A/A 10.63 and are located at the caudal tip of the neural tube. These cells clearly correspond to the *Ci-Syn *positive ones observed in tail and complete the pattern of *Ci-Syn *expression in mid tailbud embryos. Moreover, in embryos up to this stage, all post-mitotic cells are *Ci-Syn *positive and all *Ci-Syn *positive cells are post mitotic. Therefore, *Ci-Syn *expression can be considered an early marker of neuronal specification.

On the contrary, amphioxus, as well as vertebrates, do not possess this type of determinate cell lineage. More recently, the marker of cell division 5-bromo-2'-deoxyuridine (BrdU) has been used in amphioxus to assess developmental cell proliferation, showing that only some regions of the neural plate are BrdU labeled during neurogenesis [44]. In particular in mid-neurulae, only a region in the most anterior and posterior-third of the neural plate contained proliferating cells. During the subsequent phase of development, few dividing cells were present in the middle of cerebral vesicle at level of the neuropore and in some scattered cells along the posterior neural tube. A comparison between expression patterns of *AmphiSyn *and BrdU data suggests that during CNS development BrdU labeled cells correspond to the *AmphiSyn*-negative zone. Therefore, we can argue that *AmphiSyn *is a marker for postmitotic cells that have been committed to a neuronal phenotype. Although at present it is not possible to establish where the first neurons to be born are located, the most interesting observation is that the earlier expression of *AmphiSyn *corresponded to the region of the neural plate where the locomotory control system will develop. In such region, several interneurons and motor neurons have been demonstrated to be cholinergic [45]. Such assumptions are quite intriguing when compared with tunicates, in which, as above stated, the first neurons which differentiate are cholinergic. Our results shown that *AmphiSyn *and *Ci-Syn *are restricted to the post-mitotic phase of CNS development and probably are good markers of postmitotic neurons.

### *AmphiSyn *expression in the CNS

The central nervous system of amphioxus lacks an anatomical partition into fore-, mid- and hind-brain regions although several studies suggested the presence of specific anatomical domains of vertebrate brains [46-48]. The amphioxus CNS consists of a cerebral vesicle (the swelling at the anterior end of the nerve cord), corresponding to a diencephalic forebrain and a possible midbrain region, and a posterior hindbrain followed by a spinal cord.

From an evolutionary point of view, the most significant observation of the earlier *AmphiSyn *expression is its segmental organization into the area in which the hindbrain region develops. On the other hand, the expression in amphioxus embryos of motor neuron markers (such as *islet, shox, AmphiKrox, AmphiMnx *and *AmphiErr*) suggests the occurrence of a segmental organization of cell types in the hindbrain region [49-51]. Therefore, even if the amphioxus hindbrain homologous lacks a clear anatomical subdivision in rhombomeres, the segmental expression of several genes in such region seems to be regulated by adjacent somites as proposed by Mazet and Shimeld [52]. The importance of the somites for segmental neuron organization during amphioxus neurogenesis is also confirmed by the present work. In fact, *AmphiSyn *expression shows a clear segmental arrangement with cell pairs at the junctions between somites, at least at the early stage of neuronal differentiation.

At larval stage, *AmphiSyn *expression was confined in some very anterior cells of the cerebral vesicle, likely photoreceptor cells of the frontal eye complex, and in cells of the posterior cerebral vesicle. In the latter, a zone of motor neurons and large interneurones also known as primary motor centre (PMC), has been described. The PMC has been homologized to the reticulospinal neurons in the midbrain and hindbrain of craniates [53], and probably represents the region where neurotransmission via chemical synapses is most abundant [54].

The present data on *AmphiSyn *expression seems also to support the absence of typical chemical synapses in the central region of cerebral vesicle. The staining gap in the cerebral vesicle corresponds to the region where the preinfundibular region is found. The preinfundibular region, a set of ciliated accessory cells, lacks typical chemical synapses, and a paracrine release has been postulated as the preferential modality of transmission [54].

*AmphiSyn*-labeled cells were also identified more widely in the posterior larval nerve cord in the hindbrain-like region. Such cells are arranged as rows positioned ventrolaterally in the nerve cord, representing both motor neurons and interneurons. Several types of neurons in this region have been demonstrated to be cholinergic [45]. Few labeled cells located dorsally in the nerve cord are mainly found posterior to the first dorsal ocellus, except for some very anterior cells located in the posterior cerebral vesicle at the level of the lamellar body. In the latter, dopaminergic neurons have been described [55].

### *AmphiSyn *expression in PNS

*AmphiSyn *expression appears in the general ectoderm in single cells scattered along the body of the mid-neurulae and its expression increases during the elongation of the embryo. These cells probably correspond to the epidermal sensory neurons belonging to the PNS. The PNS of amphioxus is composed of several types of neurons, and at least two types of epidermal sensory cells, widely distributed along either sides of the body, have been described [56-58]. The type I receptors are primary sensory neurons having axons projecting to CNS, whereas the type II receptors are axonless secondary sensory neurons with synaptic terminals arising at short distances from the cell body. The former begin to differentiate at neurula stage while the latter start differentiation at early metamorphic larval stage. In amphioxus, differentiating type I receptors express several neuronal markers (*AmphiTrk, AmphiHu/Elav, AmphiCoe, AmphiPOU-IV, SoxB, AmphiTlx*) [59-64], as well as *AmphiSyn*.

In mid-neurulae, *AmphiSyn*-labeled cells first appeared as single epidermal cells located ventro-laterally. The cross sections revealed that most of labeled cells are found in the space between the epidermis and the deeper tissue layer. In some cases these cells exhibit a round shape, whilst in others they are elongated, probably indicating two distinct phases of sensory neuron differentiation.

The appearance of new *AmphiSyn*-expressing cells in the more dorsal position of the animal body in later neurula stages, strictly resembles the expression pattern of *Amphi-Trk*, *AmphiHu/Elav *and *AmphiCoe *[59-61]. The migration of the positive signal from ventral regions to the dorsal area and from the external layer to the subepidermal one during development, raises the question of whether this effect is due to cell migration or represents a gene expression shift. A first attempt to demonstrate such hypothesis was tentatively proved by DiI labeling experiments on amphioxus, showing that ventrally-located cells migrate toward dorso-lateral sides of the embryo [59]. More recently, the origin, migration, and differentiation of type I receptors in amphioxus have been described by SEM investigation and expression studies of *AmphiTlx *gene [64]. Such data support the idea that primary sensory neurons, developing directly from the ventral epidermis, migrate into the subepidermal sheet. During migration from ventral to dorso-lateral position, cells morphology changes: firstly round cells located ventrally lose the cilium and extend pseudopodia, subsequently elongated cells that have reached the flanks of the body give rise to an axon before reinserting their perikaria in the epidermal sheet. *AmphiTlx *is the first gene that shows clearly nascent type 1 receptors in the process of delaminating from the general epidermis and entering the subepidermal space. However, *AmphiTlx *expression is downregulated before receptor cell erupts in the epithelium.

*AmphiSyn *gene is interesting because it turns on moderately early in the migrating sensory neurons and evidently continues to be transcribed during axogenesis (and perhaps during reinsertion of the perikaria into the epidermis). On the other hand, clear examination of cross sections of the same embryo (mid-neurulae and late-neurulae) showed *AmphiSyn-*positive cells located either in the epidermis or just beneath it. Therefore, precursors of type I receptors begin to express *AmphiSyn *as they start to migrate and continue to strongly express the gene also after their complete differentiation.

Another interesting point is the observation that, in late neurulae, *AmphiSyn*-labeled cells are in register with the somites. Such arrangement is also visible in the SEM specimens reported by Kaltenbach and coworkers [64], where the migrating sensory cells appear situated at an indentation of epidermal cells at the level of intersomitic junctions. Thus, it is possible that the direction of cell migration is controlled by the somite borders. However, more work needs to be done to elucidate the patterning of amphioxus type I receptors in relation to the mesoderm.

In early larvae, the number of epidermal *AmphiSyn*-labeled cells increases in the more dorsal middle of the body, as well as in more posterior and anterior regions. In the rostral region, two large cell bodies lying at some distance from the mouth, just behind and below the preoral pit, are strongly labeled. Such cells belong to the oral nerve plexus surrounding the amphioxus larva mouth [57]. The expression pattern in the epidermal cells also persists in later stages of larval development in individual cells arranged as rows along the midline of the flanks of the body. At this stage, expression is also visible in the most anterior tip of the larva in the presumptive primary sensory neurons belonging to the corpuscles of de Quatrefages [65], structures having some similarities to the vertebrate olfactory placodes.

### Expression of *Ci-Syn *in CNS and PNS

In late tailbud embryos *Ci-Syn *is broadly expressed in all the prospective central nervous system, as the number of mature neurons increases and the single cells can not be identified any longer. In the larva, *Ci-Syn *transcripts are present in both CNS and PNS. In the sensory vesicle, *Ci-Syn *expression is present in all major clusters of neurons, including the photoreceptor cells, the eminens cells and the coronet cells. These cells have bulbous protrusions extending in the lumen of the sensory vesicle, express *Ci-TH *and were suggested to be related to the vertebrate hypothalamic primordium [66]. *Ci-Syn *transcripts were also found in the area of the otolith, which seems to be a static mechanoreceptor.

*Ci-Syn *transcripts are completely absent in the most anterior part of the sensory vesicle (anterior to the otolith), where only glial cells have been described [67], and are particularly abundant in the posterior region. This region has been recently named "posterior brain" (PB) by Horie and coworkers [32] to distinguish it from the anterior region consisting of the ventricular cavity and surrounding cells, including sensory organs. The PB contains a number of diverse interneurons, some of which form synaptic connections within the PB, and others send projections to the visceral ganglion. They presumably form neural circuits for sensory information processing and motor regulation, since many sensory neurons send axons that gather in the PB [33,68]. The widespread expression of *Ci-Syn *is a further evidence of the large amount of neuronal connections present in this area. In histological sections, the distribution of *Ci-Syn *transcripts in this area is characteristically uneven and probably labels specific subpopulations of neurons whose function is still to be investigated. Notably, in this area several types of chemically-identified neuronal types have been reported, including glutamatergic [69], cholinergic [43] and GABAergic neurons [35]. To account for the high number of neurons described in this region, neurons may be present also in *Ci-Syn*-negative areas. The expression of synaptotagmin, another synaptic vesicle protein expressed in the ascidian nervous system [33], in later stage is partially overlapping with the synapsin one, particularly in the sensory vesicle and the visceral ganglion, but it is detectable also in earlier stages. At the larva stage, synaptotagmin is widely expressed in the sensory vesicle, in some visceral ganglion motor neurons, in all the antero-trunkal epidermal neurons as well as in the caudal epidermal neurons (CEN). Thus, it is possible that a differential distribution of synapse-specific proteins exists in the PNS. In fact, the ARTENa and ARTENp, some ventral RTEN and the CEN do not express *Ci-Syn*, but they are synaptotagmin-positive. We can suppose that synaptotagmin, as others synaptic proteins, should contribute to similar function during development in different cells types and nervous regions.

At larval stage, the most interesting data show the presence in the nerve cord of *Ci-Syn *in the cell soma of the planate neurons, near the base of the tail as well as along its length. These neurons are monopolar with elongated somata aligned along the trajectory of the ventrolateral nerve bundles, whose axon projects in either caudal or rostral direction, and the most distal of them lay halfway along the tail [33]. This is consistent with our finding, since *Ci-Syn *expression reaches the level of bipolar epidermal neurons in the middle of the tail.

### Protochordate and vertebrate synapsins: a comparative overview

Synapsins are expressed throughout the central and peripheral nervous systems, and their expression varies across synapse types and brain regions [70-73]. In mammals, synapsins I and II are expressed predominantly in adult brain, whereas synapsin III appears more abundant in the fetal brain [9]. Moreover, synapsin III is required for initial cell elongation and early axon outgrowth [17]. *AmphiSyn *and *Ci-Syn *are both expressed in CNS starting at the neurula stage, where they seem to have a role in neuronal differentiation. In particular, the early expression of *AmphiSyn *and *Ci-Syn *in the developing motor neurons suggests a role for these genes in the formation of neuromuscular synapses, as already demonstrated for synapsins I and II in the *Xenopus *embryo [74,12]. Moreover, human synapsin I has been found in afferent nerve calyces surrounding type I hair cells and in sensory endings of taste buds [75-77]. At the same time, synapsins in protochordates are expressed in PNS, namely in specific subpopulations of general (non-CNS) ectoderm, representing primary sensory neurons.

In mammals, synapsin I and II are not expressed in the ribbon synapses of the retina [78], and in some invertebrates, synapsins seem to be present only in interneurons of the visual system [79]. On the contrary, both amphioxus and *Ciona *show a high concentration of synapsin transcripts in different types of the photoreceptive system, i.e. the ocellus-related cells in ascidian and the frontal eye complex and Hesse organ in amphioxus. On the other hand, the vertebrate retina is constituted by two distinct types of vesicle-containing synapses: conventional synapses, found in amacrine cells, and ribbon synapses (peculiar of vertebrates), found in photoreceptor and bipolar cells. Mandell and coworkers [78] described in several vertebrate species the absence of synapsins I and II immunoreactivities from all retina ribbon-synapses, but a high abundance of synapsin proteins in the conventional synapses of amacrine cells. However, synapsins have been identified in both cone and rod cells of bovine retina [80], suggesting that the complement of vesicle-associated proteins can vary between species.

Moreover, there are further CNS structures of amphioxus and *Ciona *having a similar distribution of synapsin transcripts, and such structures show a certain degree of similarity. For example, the sensory vesicle of the ascidian has been considered the counterpart of the cerebral vesicle of amphioxus. In particular, the otolith and ocellus are homologized with the balance organ and the lamellar body of amphioxus larvae, a clear parallel has also been suggested between the visceral ganglion of tunicate and the primary motor centre of amphioxus larva [81].

## Conclusions

Herein, we assembled and analyzed experimentally cloned and computationally predicted amino acid sequences of the synapsin genes from mammals, protochordates and several invertebrate species. Using this set of sequences, we report: (i) the occurrence of a single synapsin gene in basal metazoans and protochordates; (ii) the identification of a highly conserved genomic organization of synapsin locus; (iii) the description of the conserved domains in the synapsin protein during evolution; (iv) the protochordate synapsin expression is highly regulated during neurogenesis and nervous system formation.

## Methods

### Animal collection and RNA preparation

Amphioxus adults (*B. floridae*) were collected from Tampa Bay, FL and electrostimulated to induce gamete release. Eggs were fertilized, and embryos were cultured and fixed according to the methods of Holland and coworkers [82]. Ascidian adults (*C. intestinalis) *were collected in the bay of Naples, Italy, and reared in aquaria. Gametes were used for *in vitro *fertilization and fertilized eggs were raised in filtered sea water at 18°C. Embryos at appropriate stages were collected by low speed centrifugation and were frozen for RNA extraction or fixed for whole-mount *in situ *hybridization. Total RNA from *Ciona *larvae was extracted using the TRIzol LS reagent (Invitrogen, San Diego, CA). Following extraction, RNA was treated with RNAse-free DNAse I (Ambion Europe Ltd, UK) according to the manufacturer's recommendations in order to digest contaminating genomic DNA. First-strand cDNA was synthesized with 5 μg RNA using the SuperScript first-strand synthesis system (Invitrogen, San Diego, CA) and oligo(dT) primers.

### Amplification and sequencing of synapsin mRNAs from amphioxus and *Ciona*

The *B. floridae *and *C. intestinalis *genome sequence databases reported at JGI [83] were examined with vertebrate and invertebrate synapsin sequences using the tBlastn algorithm [84] to identify candidate gene fragments. The preliminary annotations reported at JGI [83] were carefully checked and extensively modified merging the initial sequence data to protein predictions obtained from GenScan [85] and GenomeScan [86] programs. Then, the predicted coding sequences of the synapsins were used to design gene specific primers for PCR. We used the following primers: for amphioxus, 5' primer ATGAATTATTTACGACGACGA and 3' primer CTAGGGCTCTCCGAAGATTCC-3'; for *Ciona*, 5' primer CTAAAATAAAGATGTCTTCAATGC and 3' primer ATATTCGCGCTATTGCAAGC. An embryonic cDNA library (kindly provided by Jim Langeland) was used to isolate amplicons in amphioxus, whereas in *Ciona *the amplicons were identified by RT-PCR on RNA samples from larvae. PCRs were carried out in a 50 μl reaction mixture using the Hot Master mix in accordance with the manufacturer's instructions (Eppendorf Srl, Italy). The PCR products were directly cloned using a TOPO TA cloning kit (Invitrogen, San Diego, CA) and then sequenced using a 377 PerkinElmer sequencer. During the sequencing analysis, we identified two alternative synapsin transcripts in amphioxus and a single transcript in *Ciona*. The isolated sequences were deposited at the Genbank under the following accession numbers: FJ479642 (*AmphiSyn-long*), FJ479643 (*AmphiSyn-short*) and FJ479644 (*Ci-Syn*).

### Sequence identification and genomic reconstruction

To identify synapsin gene homologues, the complete sequences of human synapsin genes were used to probe the genome of *Nematostella vectensis*, *Strongylocentrotus purpuratus*, *Capitella capitata, Drosophila melanogaster *and *Caenorhabditis elegans *using tBlastn (see Additional file 1). *Nematostella *and *Capitella *genome assemblies and protein sets were downloaded from the JGI [83]. The *S. purpuratus *assembly Spur_v1.5 and GLEAN3 gene models were obtained from Baylor College of Medicine HGSC [87]. The other genome sequences and corresponding protein sets were downloaded from Ensembl [88], NCBI [89], Trace NCBI [90] and WormBase [91] (see Additional files 1 and 5). Most of the identified sequences were annotated but many are fragmented. Where possible, these sequences were further refined by cross-reference to EST databases and direct searching, and analysis of genomic sequences. In particular, the predicted sequences on genomic browser were analyzed by GenScan [85] and GenomeScan [86] in order to correct the preliminary annotation. Exon-intron organization was reconstructed using two genomic mapping software programs: GMAP and Wise2 [92,93]. A list of sequences used in this work are provided in the Additional file 7.

### Sequence protein analysis

The Vector NTI Suite (version 9.0, Informax, North Bethesda, MD) software package was used for sequence analysis. The presence of domains and functional sequence patterns were scanned using MotifScan and InterProScan. Phosphorylation sites were predicted by using PredPhospho [94] and NetPhosK 1.0 [95]. The sequence data were also analyzed using the MEGALIGN program from LASERGENE (DNASTAR, Madison, WI) in order to evaluate sequence identity in the A, C and E domains from several invertebrate and vertebrate species.

### Phylogenetic analysis

Amino acid sequences of A and C domains and a short region of E domain from all genera were aligned using ClustalW [96]. Alignments were checked, and gaps were removed manually before construction of the trees. The alignment is shown in the Additional file 8. The maximum likelihood (ML) tree was estimated using PhyML [97,98] under the RtRev model, gamma distribution and with parameter values indicated by ProtTest [99] using the Akaike Information Criterion (AIC). The Neighbor joining (NJ) tree was obtained under the RtRev model, gamma distribution and Poisson model for amino acid evolution using MEGA 4.0 [100]. Bootstrap confidence limits were obtained by 1000 replicates in both ML and NJ analysis. Tree files were viewed by using MEGA 4.0.

### *In situ *hybridization and histological analysis

The cDNAs, corresponding to the clones *AmphiSyn-long *and *Ci-Syn *isolated by PCR, were used as templates for *in vitro *transcription by using Boehringer Mannheim DIG RNA labeling Kit, according to the supplier's instruction. *In situ *hybridization analysis on *Ciona *and amphioxus embryos was done according to the protocol described by Gionti and coworkers [101] and Holland and coworkers [82], respectively. In amphioxus, the *AmphiSyn *riboprobe is supposed to recognize both short and long transcripts. Labeled whole mount embryos were photographed using an Olympus IX71 microscope (Olympus Italia Srl, Italy), and then counterstained with 1% Ponceau S in 1% acetic acid, dehydrated in ethanol, embedded in Spurr's resin, and serially sectioned at 3-4 μm. Negative control experiments were done using sense riboprobes and no specific signal was obtained. *Ciona *whole mount embryos were also mounted with Vectashield mounting medium with DAPI (Vectastain; Vector Laboratories, Burlingame, CA).

## Authors' contributions

SC carried out the bioinformatic and molecular analysis and *in situ *hybridization assays and drafted the manuscript. RP contributed to the whole mount *in situ *experiments and to drafting the manuscript. LM contributed to the molecular analysis. FB was involved in the analysis of the conservation of synapsin domains and contributed to data discussion. FD and MP were responsible for the cellular and evolutionary interpretation of the data. All authors read and approved the final manuscript.

## Supplementary Material

Additional file 1 **Identification of the metazoans synapsin and TIMP sequences used in this study**. Identification, accession numbers and/or protein ID, genomic locations of synapsin and TIMP sequences identified in several metazoan phyla. Click here for file

Additional file 2 **Experimentally-identified and putative phosphorylation sites by various protein kinases**. The numbering of amino acids residues is based on the alignment shown in Additional file 3. Experimentally-identified phosphorylation sites are highlighted in grey. The other putative phosphorylation sites were predicted by sequence analysis using the following phosphorylation consensus sequences for the various kinases: PKA, R-R/K-X-S/T; CAMK, R-X-X-S/T; MAPK/ERK, P-X-S/T-P; cdk, S/T-P-X-R/K; PKC, S/T-X-R/K; GRK, (D/E)_n_-S/T. The synapsin isoforms are shown on the left while the phosphorylation sites 1-8 and A-Y with the respective kinases are reported in columns. Click here for file

Additional file 3 **Multiple alignments of the synapsin domains**. This file contains multiple alignments of synapsin domains performed by the AlignX program of Vector NTI. Domains A and B are indicated by azure and pink bars. The C-domain and the ATP-binding domain are indicated by yellow and grey bars, respectively. Domains D and E are indicated by red and green bars. Phosphorylation sites corresponding to sites from 1 to 8 (Ser^9^, Ser^568^, Ser^605^, Ser^62^, Ser^67^, Ser^551^, Ser^553 ^and Tyr^301^) in human synapsin I are indicated. The letters A-E, X and Y indicate the new predicted phosphorylation sites. Amino acid residues involved in ATP-binding are indicated by arrowheads. Identical and similar residues in at least 50% of the species are indicated in red and grey, respectively. Amino acid positions are numbered on the right. Taxa are abbreviated as follows: Nv, *Nematostella vectensis*, Ce, *Caenorhabditis elegans*; Dm, *Drosophila melanogaster*; Ac, *Aplysia californica*; Lp, *Loligo pealei*; Hp, *Helix pomatia*; Cc, *Capitella sp.*, Sp, *Strongylocentrotus purpuratus*; Amphi, amphioxus (*Branchiostoma floridae*); Ci, *Ciona intestinalis*; Hs, *Homo sapiens*. Click here for file

Additional file 4 **Sequence identity matrix of synapsin domains**. Comparisons of domains A, C and E from synapsins of various species after alignment using GeneWorks (Clustal W method with PAM250 weighting and identical gap costs). Colored boxes indicate percent identity of A domain (in red), C domain (in green) and E domain (in yellow). Click here for file

Additional file 5 **Other synapsin sequences used in this study**. Identification of synapsin-related sequences in two cnidarians (*Hydra magnipapillata *and *Acropora millepora*) and two protozoans (*Naegleria gruberi *and *Entamoeba histolytica*). Click here for file

Additional file 6 **Multiple alignment of *Nematostella*, amphioxus, *Ciona*, human and protozoa synapsins**. This file contains a multiple alignment of C-domain of amphioxus, Ciona, human synapsin and synapsin full-length sequences of *Naegleria gruberi *and *Entamoeba histolytica.* Click here for file

Additional file 7 **List of all the sequences used in our study**. This file contains the used protein datasets and abbreviation for the taxa. Click here for file

Additional file 8 **Sequence alignment used in the phylogenetic analysis of metazoans synapsin proteins**. This file contains the alignment generated by ClustalW in fasta format for reconstructing the phylogenetic trees of metazoan synapsins. Click here for file

## References

[B1] Huttner WB Schiebler W Greengard P De Camilli P Synapsin I (protein I), a nerve terminal-specific phosphoprotein. III. Its association with synaptic vesicles studied in a highly purified synaptic vesicle preparation J Cell Biol 1983 96 1374 1388 10.1083/jcb.96.5.1374 6404912 PMC2112660

[B2] De Camilli P Benfenati F Valtorta F Greengard P The synapsins Annu Rev Cell Biol 1990 6 433 460 10.1146/annurev.cb.06.110190.002245 1980418

[B3] Greengard P Valtorta F Czernik AJ Benfenati F Synaptic vesicle phosphoproteins and regulation of synaptic function Science 1993 259 780 785 10.1126/science.8430330 8430330

[B4] Rosahl TW Geppert M Spillane D Herz J Hammer RE Malenka RC Südhof TC Short-term synaptic plasticity is altered in mice lacking synapsin I Cell 1993 75 661 670 10.1016/0092-8674(93)90487-B 7902212

[B5] Rosahl TW Spillane D Missler M Herz J Selig DK Wolff JR Hammer RE Malenka RC Südhof TC Essential functions of synapsins I and II in synaptic vesicle regulation Nature 1995 375 488 493 10.1038/375488a0 7777057

[B6] Li L Chin LS Shupliakov O Brodin L Sihra TS Hvalby O Jensen V Zheng D McNamara JO Greengard P Impairment of synaptic vesicle clustering and of synaptic transmission, and increased seizure propensity, in synapsin I-deficient mice Proc Natl Acad Sci USA 1995 92 9235 9239 10.1073/pnas.92.20.9235 7568108 PMC40959

[B7] Chi P Greengard P Ryan TA Synapsin dispersion and reclustering during synaptic activity Nat Neurosci 2001 4 1187 1193 10.1038/nn756 11685225

[B8] Kao HT Porton B Hilfiker S Stefani G Pieribone VA DeSalle R Greengard P Molecular evolution of the synapsin gene family J Exp Zool 1999 285 360 377 10.1002/(SICI)1097-010X(19991215)285:4<360::AID-JEZ4>3.0.CO;2-3 10578110

[B9] Porton B Kao HT Greengard P Characterization of transcripts from the synapsin III gene locus J Neurochem 1999 73 2266 2271 10.1046/j.1471-4159.1999.0732266.x 10582583

[B10] Sudhof TC The synaptic vesicle cycle Annu Rev Neurosci 2004 27 509 547 10.1146/annurev.neuro.26.041002.131412 15217342

[B11] Han HQ Nichols RA Rubin MR Bahler M Greengard P Induction of formation of presynaptic terminals in neuroblastoma cells by synapsin IIb Nature 1991 349 697 700 10.1038/349697a0 1899916

[B12] Valtorta F Iezzi N Benfenati F Lu B Poo MM Greengard P Accelerated structural maturation induced by synapsin I at developing neuromuscular synapses of *Xenopus laevis* Eur J Neurosci 1995 7 261 270 10.1111/j.1460-9568.1995.tb01062.x 7757263

[B13] Sugiyama T Shinoe T Ito Y Misawa H Tojima T Ito E Yoshioka T A novel function of synapsin II in neurotransmitter release Mol Brain Res 1995 85 133 143 10.1016/S0169-328X(00)00231-X 11146115

[B14] Ferreira A Kosik KS Greengard P Han HQ Aberrant neurites and synaptic vesicle protein deficiency in synapsin II-depleted neurons Science 1994 264 977 979 10.1126/science.8178158 8178158

[B15] Ferreira A Han HQ Greengard P Kosik KS Suppression of synapsin II inhibits the formation and maintenance of synapses in hippocampal culture Proc Natl Acad Sci USA 1995 92 9225 9229 10.1073/pnas.92.20.9225 7568106 PMC40957

[B16] Chin LS Li L Ferreira A Kosik KS Greengard P Impairment of axonal development and of synaptogenesis in hippocampal neurons of synapsin I-deficient mice Proc Natl Acad Sci USA 1995 92 9230 9234 10.1073/pnas.92.20.9230 7568107 PMC40958

[B17] Ferreira A Kao HT Feng J Rapoport M Greengard P Synapsin III: developmental expression, subcellular localization, and role in axon formation J Neurosci 2000 20 3736 3744 10804215 10.1523/JNEUROSCI.20-10-03736.2000 PMC6772681

[B18] Ferreira A Rapoport M The synapsins: beyond the regulation of neurotransmitter release Cell Mol Life Sci 2002 59 589 595 10.1007/s00018-002-8451-5 12022468 PMC11337460

[B19] Pieribone VA Porton B Rendon B Feng J Greengard P Kao HT Expression of synapsin III in nerve terminals and neurogenic regions of the adult brain J Comp Neurol 2002 454 105 114 10.1002/cne.10417 12412137

[B20] Kao HT Porton B Czernik AJ Feng J Yiu G Haring M Benfenati F Greengard P A third member of the synapsin gene family Proc Natl Acad Sci USA 1998 95 4667 4672 10.1073/pnas.95.8.4667 9539796 PMC22548

[B21] Delsuc F Brinkmann H Chourrout D Philippe H Tunicates and not cephalochordates are the closest living relatives of vertebrates Nature 2006 439 965 968 10.1038/nature04336 16495997

[B22] Meinertzhagen IA Okamura Y The larval ascidian nervous system: the chordate brain from its small beginnings Trends Neurosci 2001 24 401 410 10.1016/S0166-2236(00)01851-8 11410271

[B23] Wada H Satoh N Patterning the protochordate neural tube Curr Opin Neurobiol 2001 11 16 21 10.1016/S0959-4388(00)00168-9 11179867

[B24] Klagges BR Heimbeck G Godenschwege TA Hofbauer A Pflugfelder GO Reifegerste R Reisch D Schaupp M Buchner S Buchner E Invertebrate synapsins: a single gene codes for several isoforms in *Drosophila* J Neurosci 1996 16 3154 3165 8627354 10.1523/JNEUROSCI.16-10-03154.1996 PMC6579133

[B25] Hilfiker S Schweizer FE Kao HT Czernik AJ Greengard P Augustine GJ Two sites of action for synapsin domain E in regulating neurotransmitter release Nat Neurosci 1998 1 29 35 10.1038/229 10195105

[B26] Angers A Fioravante D Chin J Cleary LJ Bean AJ Byrne JH Serotonin stimulates phosphorylation of *Aplysia *synapsin and alters its subcellular distribution in sensory neurons J Neurosci 2002 22 5412 5422 12097493 10.1523/JNEUROSCI.22-13-05412.2002 PMC6758201

[B27] Fiumara F Milanese C Corradi A Giovedì S Leitinger G Menegon A Montarolo PG Benfenati F Ghirardi M Phosphorylation of synapsin domain A is required for post-tetanic potentiation J Cell Sci 2007 120 3228 3237 10.1242/jcs.012005 17726061 PMC3016615

[B28] Hilfiker S Pieribone VA Czernik AJ Kao HT Augustine GJ Greengard P Synapsins as regulators of neurotransmitter release Philos Trans R Soc Lond B Biol Sci 1999 354 269 79 10.1098/rstb.1999.0378 10212475 PMC1692497

[B29] Onofri F Messa M Matafora V Bonanno G Corradi A Bachi A Valtorta F Benfenati F Synapsin phosphorylation by SRC tyrosine kinase enhances SRC activity in synaptic vesicles J Biol Chem 2007 282 15754 15767 10.1074/jbc.M701051200 17400547

[B30] Jovanovic JN Sihra TS Nairn AC Hemmings HC Jr Greengard P Czernik AJ Opposing changes in phosphorylation of specific sites in synapsin I during Ca^2+^-dependent glutamate release in isolated nerve terminals J Neurosci 2001 21 7944 7953 11588168 10.1523/JNEUROSCI.21-20-07944.2001 PMC6763853

[B31] Esser L Wang CR Hosaka M Smagula CS Südhof TC Deisenhofer J Synapsin I is structurally similar to ATP-utilizing enzymes EMBO J 1998 17 977 984 10.1093/emboj/17.4.977 9463376 PMC1170447

[B32] Horie T Nakagawa M Sasakura Y Kusakabe TG Cell type and function of neurons in the ascidian nervous system Dev Growth Differ 2009 51 207 220 19379276 10.1111/j.1440-169X.2009.01105.x

[B33] Imai JH Meinertzhagen IA Neurons of the ascidian larval nervous system in *Ciona intestinalis*: I. Central nervous system J Comp Neurol 2007 501 316 334 10.1002/cne.21246 17245701

[B34] Takamura K Nervous network in larvae of the ascidian *Ciona intestinalis* Dev Genes Evol 1998 208 1 8 10.1007/s004270050147 9518519

[B35] Zega G Biggiogero M Groppelli S Candiani S Oliveri D Parodi M Pestarino M De Bernardi F Pennati R Developmental expression of glutamic acid decarboxylase and of gamma-aminobutyric acid type B receptors in the ascidian *Ciona intestinalis* J Comp Neurol 2008 506 489 505 10.1002/cne.21565 18041772

[B36] Holland PW Garcia-Fernandez J Williams NA Sidow A Gene duplications and the origins of vertebrate development Dev Suppl 1994 125 133 7579513

[B37] Pohar N Godenschwege TA Buchner E Invertebrate tissue inhibitor of metalloproteinase: structure and nested gene organization within the synapsin locus is conserved from *Drosophila *to human Genomics 1999 57 293 296 10.1006/geno.1999.5776 10198170

[B38] King N Westbrook MJ Young SL Kuo A Abedin M Chapman J Fairclough S Hellsten U Isogai Y Letunic I Marr M Pincus D Putnam N Rokas A Wright KJ Zuzow R Dirks W Good M Goodstein D Lemons D Li W Lyons JB Morris A Nichols S Richter DJ Salamov A Sequencing JG Bork P Lim WA Manning G Miller WT McGinnis W Shapiro H Tjian R Grigoriev IV Rokhsar D The genome of the choanoflagellate Monosiga brevicollis and the origin of metazoans Nature 2008 451 783 788 10.1038/nature06617 18273011 PMC2562698

[B39] Watanabe H Fujisawa T Holstein TW Cnidarians and the evolutionary origin of the nervous system Dev Growth Differ 2009 51 167 183 19379274 10.1111/j.1440-169X.2009.01103.x

[B40] Sulston JE Schierenberg E White JG Thomson JN The embryonic cell lineage of the nematode *Caenorhabditis elegans* Dev Biol 1983 100 64 119 10.1016/0012-1606(83)90201-4 6684600

[B41] Yamada A Nishida H Distinct parameters are involved in controlling the number of rounds of cell division in each tissue during ascidian embryogenesis J Exp Zool 1999 284 379 391 10.1002/(SICI)1097-010X(19990901)284:4<379::AID-JEZ4>3.0.CO;2-8 10451415

[B42] Cole AG Meinertzhagen IA The central nervous system of the ascidian larva: mitotic history of cells forming the neural tube in late embryonic *Ciona intestinalis* Dev Biol 2004 271 239 262 10.1016/j.ydbio.2004.04.001 15223332

[B43] Takamura K Egawa T Ohnishi S Okada T Fukuoka T Developmental expression of ascidian neurotransmitter synthesis genes. I. Choline acetyltransferase and acetylcholine transporter genes Dev Genes Evol 2002 212 50 53 10.1007/s00427-001-0205-0 11875658

[B44] Holland ND Holland LZ Stage- and tissue-specific patterns of cell division in embryonic and larval tissues of amphioxus during normal development Evol Dev 2006 8 142 149 10.1111/j.1525-142X.2006.00085.x 16509893

[B45] Candiani S Lacalli TC Parodi M Oliveri D Pestarino M The cholinergic gene locus in amphioxus: molecular characterization and developmental expression patterns Dev Dyn 2008 237 1399 1411 10.1002/dvdy.21541 18407548

[B46] Williams NA Holland PWH Molecular evolution of the brain of chordates Brain Behav Evol 1998 52 177 185 10.1159/000006562 9787218

[B47] Holland LZ Holland ND Chordate origins of the vertebrate central nervous system Curr Opin Neurobiol 1999 9 596 602 10.1016/S0959-4388(99)00003-3 10508734

[B48] Wicht H Lacalli TC The nervous system of amphioxus: structure, development, and evolutionary significance Can J Zool 2005 83 122 150 10.1139/z04-163

[B49] Ferrier DE Brooke NM Panopoulou G Holland PW The Mnx homeobox gene class defined by HB9, MNR2 and amphioxus *AmphiMnx* Dev Genes Evol 2001 211 103 107 10.1007/s004270000124 11455421

[B50] Jackman WR Kimmel CB Coincident iterated gene expression in the amphioxus neural tube Evol Dev 2002 4 366 374 10.1046/j.1525-142X.2002.02022.x 12356266

[B51] Bardet PL Schubert M Horard B Holland LZ Laudet V Holland ND Vanacker JM Expression of estrogen-receptor related receptors in amphioxus and zebrafish: implications for the evolution of posterior brain segmentation at the invertebrate-to-vertebrate transition Evol Dev 2005 7 223 233 10.1111/j.1525-142X.2005.05025.x 15876195

[B52] Mazet F Shimeld SM The evolution of chordate neural segmentation Dev Biol 2002 251 258 270 10.1006/dbio.2002.0831 12435356

[B53] Fritzsch B Similarities and differences in lancelet and craniates nervous systems Isr J Zool 1996 42 S147 S160

[B54] Lacalli TC Kelly SJ The infundibular balance organ in amphioxus larvae and related aspects of cerebral vesicle organization Acta Zool (Stockh) 2000 81 37 47 10.1046/j.1463-6395.2000.00036.x

[B55] Candiani S Oliveri D Parodi M Castagnola P Pestarino M *AmphiD1/beta*, a dopamine D1/beta-adrenergic receptor from the amphioxus *Branchiostoma floridae*: evolutionary aspects of the catecholaminergic system during development Dev Genes Evol 2005 215 631 638 10.1007/s00427-005-0019-6 16187137

[B56] Stokes MD Holland ND Embryos and larvae of a lancelet, *Branchiostoma floridae*, from hatching through metamorphosis: growth in the laboratory and external morphology Acta Zool (Stockh) 1995 76 105 120

[B57] Lacalli TC Hou S A re-examination of the epithelial sensory cells of amphioxus (*Branchiostoma*) Acta Zool (Stockh) 1999 80 125 134 10.1046/j.1463-6395.1999.80220005.x

[B58] Holland ND Yu JK Epidermal receptor development and sensory pathways in vitally stained amphioxus (*Branchiostoma floridae*) Acta Zool (Stockh) 2002 83 309 319 10.1046/j.1463-6395.2002.00120.x

[B59] Benito-Gutiêrrez E Nake C Llovera M Comella JX Garcia-Fernàndez J The single AmphiTrk receptor highlights increased complexity of neurotrophin signalling in vertebrates and suggests an early role in developing sensory neuroepidermal cells Development 2005 132 2191 2202 10.1242/dev.01803 15799999

[B60] Satoh G Wang Y Zhang P Satoh N Early development of amphioxus nervous system with special reference to segmental cell organization and putative sensory cell precursors: a study based on the expression of pan-neuronal marker gene Hu/elav J Exp Zool 2001 291B 354 364 10.1002/jez.1134 11754014

[B61] Mazet F Masood S Luke GN Holland ND Shimeld SM Expression of *AmphiCoe*, an amphioxus COE/EBF gene, in the developing central nervous system and epidermal sensory neurons Genesis 2004 38 58 65 10.1002/gene.20006 14994268

[B62] Candiani S Oliveri D Parodi M Bertini E Pestarino M Expression of AmphiPOU-IV in the developing neural tube and epidermal sensory neural precursors in amphioxus supports a conserved role of class IV POU genes in the sensory cells development Dev Genes Evol 2006 216 623 633 10.1007/s00427-006-0083-6 16773340

[B63] Meulemans D Broner-Fraser M The amphioxus SoxB family: implications for the evolution of vertebrate placodes Int J Biol Sci 2007 3 356 364 17713598 10.7150/ijbs.3.356 PMC1950271

[B64] Kaltenbach SL Yu JK Holland ND The origin and migration of the earliest-developing sensory neurons in the peripheral nervous system of amphioxus Evol Dev 2009 11 142 151 10.1111/j.1525-142X.2009.00315.x 19245546

[B65] Demski LS Beaver JA Morrill JB The cutaneous innervations of amphioxus: A review incorporating new observations with DiI tracing and scanning electron microscopy Isr J Zool 1996 42 117 129

[B66] Moret F Christiaen L Deyts C Blin M Joly JS Vernier P The dopamine-synthesizing cells in the swimming larva of the tunicate *Ciona intestinalis *are located only in the hypothalamus-related domain of the sensory vesicle Eur J Neurosci 2005 21 3043 3055 10.1111/j.1460-9568.2005.04147.x 15978015

[B67] Nicol D Meinertzhagen IA Cell counts and maps in the larval central nervous system of the ascidian *Ciona intestinalis *(L.) J Comp Neurol 1991 309 415 429 10.1002/cne.903090402 1918443

[B68] Tsuda M Sakurai D Goda M Direct evidence for the role of pigment cells in the brain of ascidian larvae by laser ablation J Exp Biol 2003 206 1409 1417 10.1242/jeb.00235 12624175

[B69] Horie T Kusakabe T Tsuda M Glutamatergic networks in the *Ciona intestinalis *larva J Comp Neurol 2008 508 249 263 10.1002/cne.21678 18314906

[B70] Fried G Nestler EJ De Camilli P Stjärne L Olson L Lundberg JM Hökfelt T Ouimet CC Greengard P Cellular and subcellular localization of protein I in the peripheral nervous system Proc Natl Acad Sci USA 1982 79 2717 2721 10.1073/pnas.79.8.2717 6806817 PMC346273

[B71] Südhof TC Czernik AJ Kao HT Takei K Johnston PA Horiuchi A Kanazir SD Wagner MA Perin MS De Camilli P Synapsins: mosaics of shared and individual domains in a family of synaptic vesicle phosphoproteins Science 1989 245 1474 1480 10.1126/science.2506642 2506642

[B72] Mandell JW Czernik AJ De Camilli P Greengard P Townes-Anderson E Differential expression of synapsins I and II among rat retinal synapses J Neurosci 1992 12 1736 1749 1578266 10.1523/JNEUROSCI.12-05-01736.1992 PMC6575884

[B73] Matus-Leibovitch N Nevo I Vogel Z Differential distribution of synapsin IIa and IIb mRNAs in various brain structures and the effect of chronic morphine administration on the regional expression of these isoforms Brain Res Mol Brain Res 1997 45 301 316 10.1016/S0169-328X(96)00265-3 9149105

[B74] Schaeffer E Alder J Greengard P Poo MM Synapsin IIa accelerates functional development of neuromuscular synapses Proc Natl Acad Sci USA 1994 91 3882 3886 10.1073/pnas.91.9.3882 8171006 PMC43686

[B75] Favre D Scarfone E Di Gioia G De Camilli P Dememes D Presence of synapsin I in afferent and efferent nerve endings of vestibular sensory epithelia Brain Res 1986 384 379 382 10.1016/0006-8993(86)91176-5 3096490

[B76] Finger TE Womble M Kinnamon JC Ueda T Synapsin I-like immunoreactivity in nerve fibers associated with lingual taste buds of the rat J Comp Neurol 1990 292 283 290 10.1002/cne.902920210 2108194

[B77] Scarfone E Demêmes D Sans A Synapsin I and Synaptophysin expression during ontogenesis of the mouse peripheral vestibular system J Neurosci 1991 11 1173 1181 1902873 10.1523/JNEUROSCI.11-05-01173.1991 PMC6575312

[B78] Mandell JW Townes-Anderson E Czernik AJ Cameron R Greengard P De Camilli P Synapsins in the vertebrate retina: absence from ribbon synapses and heterogeneous distribution among conventional synapses Neuron 1990 5 19 33 10.1016/0896-6273(90)90030-J 2114884

[B79] Leitinger G Pabst MA Rind FC Simmons PJ Differential expression of synapsin in visual neurons of the locust *Schistocerca gregaria* J Comp Neurol 2004 480 89 100 10.1002/cne.20333 15514920

[B80] Von Kriegstein K Schmitz F Link E Südhof TC Distribution of synaptic vesicle proteins in the mammalian retina identifies obligatory and facultative components of ribbon synapses Eur J Neurosci 1999 11 1335 1348 10.1046/j.1460-9568.1999.00542.x 10103129

[B81] Lacalli TC Frontal eye circuitry, rostral sensory pathways and brain organization in amphioxus larvae: evidence from 3D reconstructions Philosophical Transactions of the Royal Society 1996 B351 243 263 10.1098/rstb.1996.0022

[B82] Holland LZ Holland PWH Holland ND Ferraris JD Palumbi SR Revealing homologies between body parts of distantly related animals by *in situ *hybridization to developmental genes: amphioxus versus vertebrates Molecular Zoology: Advances, Strategies, and Protocols 1996 New York: Wiley-Liss 267 282

[B83] JGI (Joint Genome Institute) http://genome.jgi-psf.org/

[B84] Altschul SF Gish W Miller W Myers EW Lipman DJ Basic local alignment search tool J Mol Biol 1990 215 403 410 2231712 10.1016/S0022-2836(05)80360-2

[B85] GenScan http://genes.mit.edu/GENSCAN.html

[B86] GenomeScan http://genes.mit.edu/genomescan.html

[B87] Baylor College of Medicine HGSC http://annotation.hgsc.bcm.tmc.edu/Urchin/cgi-bin/pubLogin.cgi

[B88] Ensembl http://www.ensembl.org/index.html

[B89] NCBI http://www.ncbi.nlm.nih.gov/

[B90] Trace NCBI http://www.ncbi.nlm.nih.gov/Traces/

[B91] WormBase http://www.wormbase.org/

[B92] GMAP http://www.gene.com/share/gmap/

[B93] Wise2 http://www.ebi.ac.uk/Wise2/

[B94] PredPhospho http://www.nih.go.kr/predphospho/proteo/html/inc_PredPhospho.htm

[B95] NetPhosK 1.0 http://www.cbs.dtu.dk/services/NetPhosK/

[B96] ClustalW http://www.ebi.ac.uk/Tools/clustalw

[B97] Guindon S Gascuel O A simple, fast, and accurate algorithm to estimate large phylogenies by maximum likelihood Syst Biol 2003 52 696 704 10.1080/10635150390235520 14530136

[B98] Guindon S Lethiec F Duroux P Gascuel O PHYML Online--a web server for fast maximum likelihood-based phylogenetic inference Nucleic Acids Res 2005 33 Web Server W557 559 10.1093/nar/gki352 15980534 PMC1160113

[B99] Abascal F Zardoya R Posada D ProtTest: selection of best-fit models of protein evolution Bioinformatics 2005 12 2104 2105 10.1093/bioinformatics/bti263 15647292

[B100] Tamura K Dudley J Nei M Kumar S MEGA4: Molecular Evolutionary Genetics Analysis (MEGA) software version 4.0 Mol Biol Evol 2007 24 1596 1599 10.1093/molbev/msm092 17488738

[B101] Gionti M Ristoratore F DiGregorio A Aniello F Branno M Di Lauro R *Cihox5*, a new *Ciona intestinalis *Hox-related gene, is involved in regionalization of the spinal cord Dev Genes Evol 1998 207 515 523 10.1007/s004270050142 9510546

