# Supplementary material for: The synapsin gene family in basal chordates: evolutionary perspectives in metazoans
Source: BMC Evol Biol. 2010 Jan 29;10:32. doi: 10.1186/1471-2148-10-32 (PMC2825198; doi:10.1186/1471-2148-10-32)
Supplement: Additional file 1 — Identification of the metazoans synapsin and TIMP sequences used in this study. Identification, accession numbers and/or protein ID, genomic locations of synapsin and TIMP sequences identified in several metazoan phyla. [file 1471-2148-10-32-S1.PDF]

	SYN-TIMP LOCUS						EXTERNAL TIMP			DATABASES
**Species**	**Name**	**Genomic coordinates CDS**	**GenBank Accession or Protein ID**	**Name**	**Genomic coordinates CDS**	**GenBank Accession or Protein ID**	**Name**	**Genomic coordinates CDS**	**GenBank Accession or Protein ID**	
Homo sapiens		Xp11.23			Xp11.23					
	SYN-Ia	2485730-2438861	NP_008881	***TIMP 1***	2449413-2444526	AAH00866				NCBI (Celera assembly)
	SYN-Ib	2485730-2438791	NP_598006							
		3p25			3p25					
	SYN-IIa	11983152-12169542	NP_598328	***TIMP4***	12137762-12132490	NP_003247				NCBI (Celera assembly)
	SYN-IIb	11983152-12162360	NP_003169							
		22q12.3								
	SYN-IIIa	12793216-12300248	NP_003481							
	SYN-IIIb	12793216-12304801	predicted		22q12.3					
	SYN-IIIc	12793216-12300364	NP_598344	***TIMP3***	12588556-12645737	NP_000353				NCBI
	SYN-IIId	12793216-12651426	predicted							
	SYN-IIIe	12315561-12300248	predicted							
	SYN-IIIf	12315561-12304801	predicted							
							TIMP 2	17q25:10847442-10778021	NP_003246	NCBI
Branchiostoma floridae	***AmphiSynI/s***	scf_807:46322-88547<br>scf_37:1203341-1239246	ID: 110844<br>ID: 209019<br>FJ479642/FJ479643	***AmphiTIMPi***	scf_807:53615-70350<br>scf_37:1218195-1232233	ID: 132868<br>predicted				JGI (v1.0)
							AmphiTIMPe	scf_135:232508-228024<br>scf_480: 75007-79442	ID:84794<br>ID:187194	
Ciona intestinalis	***Ci-Syn***	scf_53:235956:260096<br>chr_05q:4350736-4326457	cieg001p09<br>ID: 260202<br>FJ479644	***CiTIMPA***	scf_53:247786-244743<br>chr_05q:4338770-4341816	ID: 139906<br>ID: 202283				JGI (v1.0/v2.0)
				CiTIMPB	scf_53: 256060-251906<br>chr_05q:4330479-4334641	ID: 139932<br>ID: 260202				
Strongylocentrotus purpuratus	***SpSyn***	scf_155: 440399-28824	predicted	***SpTIMPA***	scf155 :384940-399099<br>(LOC575539)	XM_775934<br>GLEAN3_08866				
				SpTIMPB	scf155 :336251-379053<br>(LOC753842)	XM_001177881<br>GLEAN3_08865				
				SpTIMPC	scf155 :304571-310325<br>(LOC753828)	XM_001177756<br>GLEAN3_08864				
				SpTIMPD	scf155 :192939-287613<br>(LOC575414)	XM_775817<br>GLEAN3_08863				NCBI (gnl_Spur_v1.5)<br>NW_737165<br>HGSC
				SpTIMPE	scf155 :164811-167078<br>(LOC575337)	XM_775744<br>GLEAN3_08862				
				SpTIMPF	scf155 :132644-135537<br>predicted	**GLEAN3_08861**				
				SpTIMPG	scf155 :118427-123394<br>(LOC575130)	XM_775549<br>GLEAN3_08860				
				SpTIMPH	scf155 :109540-112535<br>(LOC765493)	XM_001196571<br>GLEAN3_08859				
				<u>SpTIMPI</u>	scf155 :100961-103224	**GLEAN3_08858**				
				<u>SpTIMPL</u>	scf155 :60280-73805	**GLEAN3_08857**				
Drosophila melanogaster		chr3R			chr3R					
	DmSynA	6021091-6043446	NP_731457	***DmTIMP***	6031104-6030236	AAF54507				
	DmSynE	6021559-6043295	NP_731458							
	DmSynC	6021091-6043446	NP 731460							Ensembl
	DmSynD	6021559-6045865	NP_731459							
	DmSynF	6021091-6045865	NP_788628							
Caenorhabditis elegans		chrIV								
	Cesnn1a	1805171-1815094	NP_741327							Ensembl
	Cesnn1b	1797964-1815094	NP_741326							
							CeTIMPA	chrV:8201788-8202504	AAA96174	
							CeTIMPB	chrV:8207043-8208412	NP_505115	
Caenorhabditis briggsae		chrIV								
	Cbrsnn1a	12381070-12382623	CBP25623							WormBase
	Cbrsnn1b	12379425-12382623	predicted							
							CbrTIMPA	chrV:725170-726011	CBP02761	
							CbrTIMPB	chrV:730394-731751	CBP17165	
Caenorhabditis brenneri		contig 194								
	Cbnsnn1a	12912-12499	CN35320							WormBase
	Cbnsnn1b	14380-12499	predicted							
							CbnTIMPA	contig 85:174136-175603	predicted	
							CbnTIMPB	contig 85:182855-184400	CN16198	
Caenorhabditis remanei		contig 88								
	Cresnn1a	318413-323239	predicted							WormBase
	Cresnn1b	316777-323239	RP29804							
							CreTIMPA	contig 13:631785-632432	RP18226	
							CreTIMPB	contig 13:636337-639957	RP03961	
Caenorhabditis japonica		contig 163								
	Cjpsnn1a	97744-95651	predicted							WormBase
	Cjpsnn1b	100211-95651	JA10084							
							CjpTIMPA	contig 195:64582-63060	JA36276	
							CjpTIMPB	contig 195:58211-55332	predicted	
Brugia malayi	***Bmsnn***	scf_14972: 1026374-1021760	XP_001898864							WormBase
Capitella capitata	***CcSyn***	scf_266:159674-211375	predicted	***CcTIMP***	scf_266: 205491-207406	predicted				JGI
Nematostella vectensis	***NvSyn***	scf_99: 165180-180738	predicted<br>EST:FC258375	***NvTIMPi***	scf_99: 173998-170281	ID: 208948				
							NvTIMPAe	scf_4:1086096-1089690	ID: 238195	
							<u>NvTIMPBe</u>	scf_271:290106-293235	ID: 216776	JGI
							<u>NvTIMPCe</u>	scf_271:286474-288708	ID: 216775	
							<u>NvTIMPDe</u>	scf_11:977870-980528	ID: 198684	
							NvTIMPEe	scf_2:2101832-2102812	ID: 237953	

*underlined are the sequences not confirmed by any EST and/or cDNA. In red are the sequences cloned in this work.
