# Supplementary material for: The synapsin gene family in basal chordates: evolutionary perspectives in metazoans
Source: BMC Evol Biol. 2010 Jan 29;10:32. doi: 10.1186/1471-2148-10-32 (PMC2825198; doi:10.1186/1471-2148-10-32)
Supplement: Additional file 3 — Multiple alignments of the synapsin domains. This file contains multiple alignments of synapsin domains performed by the AlignX program of Vector NTI. Domains A and B are indicated by azure and pink bars. The C-domain and the ATP-binding domain are indicated by yellow and grey bars, respectively. Domains D and E are indicated by red and green bars. Phosphorylation sites corresponding to sites from 1 to 8 (Ser9, Ser568, Ser605, Ser62, Ser67, Ser551, Ser553 and Tyr301) in human synapsin I are indicated. The letters A-E, X and Y indicate the new predicted phosphorylation sites. Amino acid residues involved in ATP-binding are indicated by arrowheads. Identical and similar residues in at least 50% of the species are indicated in red and grey, respectively. Amino acid positions are numbered on the right. Taxa are abbreviated as follows: Nv, Nematostella vectensis, Ce, Caenorhabditis elegans; Dm, Drosophila melanogaster; Ac, Aplysia californica; Lp, Loligo pealei; Hp, Helix pomatia; Cc, Capitella sp., Sp, Strongylocentrotus purpuratus; Amphi, amphioxus (Branchiostoma floridae); Ci, Ciona intestinalis; Hs, Homo sapiens. [file 1471-2148-10-32-S3.DOC]

DOMAIN A

DOMAIN B

**1 4 5**

**NvSyn**  **------------------------MELWQKLKNTNPIAVGIRNAHERYKEEHAS-----------------------------------------------------KQTTTNGSKEDI--SKTKSPESK---------- 51**

**Cesnn1b ------------------------MNFFKRKFSFSEDEGEPMDDVNSGPPSSFSF------------------------------------------------QSIANKVSNTIS-APT--SPAKSRES----------- 54**

**DmSyna -----------------------------------------------------------------------MPPPPAPGQPA-GAAPELSLSFGAGKTPATAA-PAPPR-GV--S-APT--SPAKSRES----------- 50**

**DmSync -----------------------------------------------------------------------MPPPPAPGQPA-GAAPELSLSFGAGKTPATAA-PAPPR-GV--S-APT--SPAKSRES----------- 50**

**DmSyne -----------MNFSSFKSSFTSNVNFLKRRFSSGDLSSEVDDVDPNSLPPAARPIQDQPTKPPVAGGPPNMPPPPAPGQPA-GAAPELSLSFGAGKTPATAA-PAPPR-GV--S-APT--SPAKSRES----------- 110**

**AcSyn11.1 ------------------------MNYLRRRFSSGDLQGEASDNDDSPNVGGLN-----------------------------------------------------FRKGPSPS-APN--SPSKSASSAN--------- 51**

**AcSyn2.1 -----------MSFSNFKDSFGSGMNYLRRRFSSGDLQGEASDNDDSPNVGGLN-----------------------------------------------------FRKGPSPS-APN--SPSKSASSAN--------- 64**

**AcSyn8.2 -----------MSFSNFKDSFGSGMNYLRRRFSSGDLQGEASDNDDSPNVGGLN-----------------------------------------------------FRKGPSPS-APN--SPSKSASSAN--------- 64**

**AcSyn7.1 ------------------------MNYLRRRFSSGDLQGEASDNDDSPNVGGLN-----------------------------------------------------FRKGPSPS-APN--SPSKSASSAN--------- 51**

**LpSyn-long FFFDLFFQTAKISFSGFRDNFSTGVGFLKRRFSSGDLQGELRDAQEQGQVPILP-----------------------------------------------------IRKGPSPS-APS--SPSKTTAAGIAK------- 77**

**LpSyn-short FFFDLFFQTAKISFSGFRDNFSTGVGFLKRRFSSGDLQGELRDAQEQGQVPILP-----------------------------------------------------IRKGPSPS-APS--SPSKTTAAGIAK------- 77**

**HpSyn ------------------------MNFLRRRFSSGDLQGEANEKEDPPNVGILN-----------------------------------------------------FKKGPSPS-APN--SPSKSASPAT--------- 51**

**CcSyn ------------------------MNFLRRRFSSSDLQGELADSSAGPQAPDNSG--------------------------------------------FSFNFP---KKGPSPS-APS--SPSKSSSSVQGIT------ 60**

**SpSyn ------------------------MNYLRRRFSDPNAMTGLPDGYFQNIGQEEVV---------------------------------------------------EKRTAISTSN-----SPTMPRRDNSM-------- 52**

**AmphiSyn-long ------------------------MNYLRRRFSDTNIAANLPNGYLSGLGGEDD------------------------------------------KNQQQPAA-APPR-G-T-S-APS--SPARSMPP----------- 56**

**AmphiSyn-short ------------------------MNYLRRRFSDTNIAANLPNGYLSGLGGEDD------------------------------------------KNQQQPAA-APPR-G-T-S-APS--SPARSMPP----------- 56**

**Ci-Syn -----------------MSSMQNAMNFLKRRFSDTNFNSNLPNGYLESDDDVSVT-----------------------SSVSRG-TP--------QK--QQIAQPGINQQNKINVNPAQSGQV-SST------------- 75**

**HsSynIa ------------------------MNYLRRRLSDSNFMANLPNGYMTDLQRPQP------------------PPPP-PGAHSPGATP------GPG-T-ATAERSSGVAPAASPA-AP---SPGSSGG------------ 73**

**HsSynIb ------------------------MNYLRRRLSDSNFMANLPNGYMTDLQRPQP------------------PPPP-PGAHSPGATP------GPG-T-ATAERSSGVAPAASPA-AP---SPGSSGG------------ 73**

**HsSynIIa -----------------------MMNFLRRRLSDSSFIANLPNGYMTDLQRPEPQ--------------QPPPPPP-PG---PGAA--------------------------SASAAPPTASPGPERRPPPASAPAPQPA 73**

**HsSynIIb -----------------------MMNFLRRRLSDSSFIANLPNGYMTDLQRPEPQ--------------QPPPPPP-PG---PGAA--------------------------SASAAPPTASPGPERRPPPASAPAPQPA 73**

**HsSynIIIa ------------------------MNFLRRRLSDSSFMANLPNGYMTDLQRPDS-------------------------------------------------------STSSPA------SPAMERRHPQPLAASFSSP 55**

**HsSynIIIb ------------------------MNFLRRRLSDSSFMANLPNGYMTDLQRPDS-------------------------------------------------------STSSPA------SPAMERRHPQPLAASFSSP 55**

**HsSynIIIc ------------------------MNFLRRRLSDSSFMANLPNGYMTDLQRPDS-------------------------------------------------------STSSPA------SPAMERRHPQPLAASFSSP 55**

**NvSyn**  **-------------------------------------------KLT 54**

**Cesnn1b ----LAAALERSLNHDRSR------------------GEPLM-KDA 77**

**DmSyna ----LLQRVQ-SLTGAARDQG-ASILGAAVQSATQRAPAFSK-DKY 89**

**DmSync ----LLQRVQ-SLTGAARDQG-ASILGAAVQSATQRAPAFSK-DKY 89**

**DmSyne ----LLQRVQ-SLTGAARDQG-ASILGAAVQSATQRAPAFSK-DKY 149**

**AcSyn11.1 ----LGQRLFSSSSSSSGK------------------PSYNK-DRC 74**

**AcSyn2.1 ----LGQRLFSSSSSSSGK------------------PSYNK-DRC 87**

**AcSyn8.2 ----LGQRLFSSSSSSSGK------------------PSYNK-DRC 87**

**AcSyn7.1 ----LGQRLFSSSSSSSGK------------------PSYNK-DRC 74**

**LpSyn-long -----G--VFSGPK-----------------------ASVNK-DRC 92**

**LpSyn-short -----G--VFSGPK-----------------------ASVNK-DRC 92**

**HpSyn ----IGQKLFSGTVGV---------------------KPVSK-DRY 71**

**CcSyn ----------RSLINAAQNAATGVGGK----------AGYNK-ERC 85**

**SpSyn -----G--FLSSLA-----------------------PRQEK-GNQ 67**

**AmphiSyn-long -----GGMAGG--------------------------RDGER-NTT 70**

**AmphiSyn-short -----GGMAGG--------------------------RDGER-NTT 70**

**Ci-Syn -----GG-FFASLASGNPLAVLK--------------VDGN--EQI 99**

**HsSynIa -----GG-FFSSLSNAVKQTTAAAAATFSEQVGGGSGGAGRGGAAS 113**

**HsSynIb -----GG-FFSSLSNAVKQTTAAAAATFSEQVGGGSGGAGRGGAAS 113**

**HsSynIIa PTPSVGSSFFSSLSQAVKQTA-ASAGLVDAPAP----APAAA-RKA 113**

**HsSynIIb PTPSVGSSFFSSLSQAVKQTA-ASAGLVDAPAP----APAAA-RKA 113**

**HsSynIIIa -----GSSLFSSLSSAMKQAPQATSGLMEPPGP----STPIV-QRP 91**

**HsSynIIIb -----GSSLFSSLSSAMKQAPQATSGLMEPPGP----STPIV-QRP 91**

**HsSynIIIc -----GSSLFSSLSSAMKQAPQATSGLMEPPGP----STPIV-QRP 91**

**ATP BINDING DOMAIN**

**DOMAIN C**

**A B**

**NvSyn KTLLIIDSADHDWSKIFKGRNLHNGSYGIRVEQAEFADINLASYSDSGTMVDIQVFREGTVVV-RSFRPDFVLVRQSIRGIGPREDYRHILLGLQFGNVPSVNSLESIYNFAEKPWVFSQLIQIKKRLGKEEFPLIEQAYYPNHKEM-----LITPRFPV 208**

**Cesnn1a** **KVLLVIDSHHVDWSKYFRN---H-TEYAIRVEQGDIDELDVMC-TEKNCTVELNTP--GKD-V-RTFTPSAVFLGAGATR---CAQLKTITRAFIAAHIPFLNSHTSAVAFLDRNNLKKQLKKITLSDGA-SIPMLPIVHYPHFHKFHQ---SQSSTYPM 148**

**Cesnn1b** **KVLLVIDSHHVDWSKYFRN---H-TEYAIRVEQGDIDELDVMC-TEKNCTVELNTP--GKD-V-RTFTPSAVFLGAGATR---CAQLKTITRAFIAAHIPFLNSHTSAVAFLDRNNLKKQLKKITLSDGA-SIPMLPIVHYPHFHKFHQ---SQSSTYPM 221**

**DmSyna** **FTLLVLDDQNTDWSKYFRGRRLH-GDFDIRVEQAEFRDITVVSSADTGPVVTMAAYRSGTRVA-RSFRPDFVLIRQPPRDG--SSDYRSTILGLKYGGVPSINSLHSIYQFQDKPWVFSHLLQLQRRLGRDGFPLIEQTFFPNPRDL-----FQFTKFPS 240**

**DmSync** **FTLLVLDDQNTDWSKYFRGRRLH-GDFDIRVEQAEFRDITVVSSADTGPVVTMAAYRSGTRVA-RSFRPDFVLIRQPPRDG--SSDYRSTILGLKYGGVPSINSLHSIYQFQDKPWVFSHLLQLQRRLGRDGFPLIEQTFFPNPRDL-----FQFTKFPS 240**

**DmSyne** **FTLLVLDDQNTDWSKYFRGRRLH-GDFDIRVEQAEFRDITVVSSADTGPVVTMAAYRSGTRVA-RSFRPDFVLIRQPPRDG--SSDYRSTILGLKYGGVPSINSLHSIYQFQDKPWVFSHLLQLQRRLGRDGFPLIEQTFFPNPRDL-----FQFTKFPS 300**

**AcSyn11.1** **KTLLVIDDQHTDWSKYFRGKKLF-GDWDVRVEQAEFSELNLAAYSDSGTMVDIQVTRNGTKVV-RSFKPDFVLIRQHVRDA--HEDWRNLLLGFKYGAIPSVNSLTAEYNFLDKPWVFAQLIEIQKRLGKESFPLIDQAYYPNHKEM-----LITPKFPV 225**

**AcSyn2.1** **KTLLVIDDQHTDWSKYFRGKKLF-GDWDVRVEQAEFSELNLAAYSDSGTMVDIQVTRNGTKVV-RSFKPDFVLIRQHVRDA--HEDWRNLLLGFKYGAIPSVNSLTAEYNFLDKPWVFAQLIEIQKRLGKESFPLIDQAYYPNHKEM-----LITPKFPV 238**

**AcSyn8.2** **KTLLVIDDQHTDWSKYFRGKKLF-GDWDVRVEQAEFSELNLAAYSDSGTMVDIQVTRNGTKVV-RSFKPDFVLIRQHVRDA--HEDWRNLLLGFKYGAIPSVNSLTAEYNFLDKPWVFAQLIEIQKRLGKESFPLIDQAYYPNHKEMVSNMWLITPKFPV 243**

**AcSyn7.1** **KTLLVIDDQHTDWSKYFRGKKLF-GDWDVRVEQAEFSELNLAAYSDSGTMVDIQVTRNGTKVV-RSFKPDFVLIRQHVRDA--HEDWRNLLLGFKYGAIPSVNSLTAEYNFLDKPWVFAQLIEIQKRLGKESFPLIDQAYYPNHKEMVSNMWLITPKFPV 230**

**LpSyn-long** **KTLLVIDDPHTDWSKYFRGKKIF-GDWDLRVEQVEFHEINLAAYTDQGTMVDIQVMRNGTRVV-RSFKPDFVLVRQHVRDA--SEDWRNLIMGFHYGGIPSLNSFHSIYNFQDKPWTMAHLIQIQKKVGAENFPLIEQAYYPNHKEM-----LVTPKFPV 243**

**LpSyn-short** **KTLLVIDDPHTDWSKYFRGKKIF-GDWDLRVEQVEFHEINLAAYTDQGTMVDIQVMRNGTRVV-RSFKPDFVLVRQHVRDA--SEDWRNLIMGFHYGGIPSLNSFHSIYNFQDKPWTMAHLIQIQKKVGAENFPLIEQAYYPNHKEM-----LVTPKFPV 243**

**HpSyn KTLLVIDGQHTDWSKYFKGKKLF-GDWDVKVEQAEFSELNLASNSETGTTVEIQAIRNGNKTT-RSLKPDFLLIRQHVRDA--KVDWRHLLLGFRYGGVPSINSLTAEFNFLDKPWVFAQLIDIQKRLSKDVFPLIDQTYFSNHEEM-----LNSPKFPL 222**

**CcSyn KTLLVIDDQHTDWSKYFKGRKIF-GDWDIRIEQATFSEINLAAYTDSGTVVDIQVTRNGTKVV-RSFKPDFVLVRQHVRDA--NENWKNIILGLQYGGIPSLNSLNSIFNFLEKPWVFAHLIQIQKRLGKDRFPLIEQSYYPNHKEMVSNLWLVTPKFPV 241**

**SpSyn KTLLVIDDLHTDWAKYFRGKLIH-GEYEIRVEQCEFSELNLASYSDAGVTVDMRGIRQGQRVV-RTFKPDYVLVRQHARSMEVQEDWRNLVIGFQYGNVPSLNSWQVVYNFMDKPWV---LTTRQEKLGKEKFPLVDQAFFPNHREM-----LTTPRFPV 217**

**AmphiSyn-long KTLLVIDDQHTDWGKYFRGKRIN-NEYEIRVEQADFSEINLAAYSDTGTMIDMQINRQGTKVV-RSFRPDFVLVRQHCRGLDANQDYRSVILGLRWGAIPSVNTLLSIYNFMEKPWVYAHLLQIRKRIGKEKFPLIDRAYYPNHKEM-----LITPKFPV 223**

**AmphiSyn-short KTLLVIDDQHTDWGKYFRGKRIN-NEYEIRVEQADFSEINLAAYSDTGTMIDMQINRQGTKVV-RSFRPDFVLVRQHCRGLDANQDYRSVILGLRWGAIPSVNTLLSIYNFMEKPWVYAHLLQIRKRIGKEKFPLIDRAYYPNHKEM-----LITPKFPV 223**

**Ci-Syn KTVLVVDEQHTDWSKYLKGRKIH-NDYTVRVEQAEFSELSLSSHSVNGVTVDINCYRNGNKVV-RSFKPDFLLVRQTPRSMAQGEDFRNLVIGLKYGGIPSVNSLHSQFNFMDKPWTFSQLIRIQKRLGPEKFPLVEQTYYPSHKQM-----LTSSHFPV 252**

**HsSynIa RVLLVIDEPHTDWAKYFKGKKIH-GEIDIKVEQAEFSDLNLVAHANGGFSVDMEVLRNGVKVV-RSLKPDFVLIRQHAFSMARNGDYRSLVIGLQYAGIPSVNSLHSVYNFCDKPWVFAQMVRLHKKLGTEEFPLIDQTFYPNHKEM-----LSSTTYPV 266**

**HsSynIb RVLLVIDEPHTDWAKYFKGKKIH-GEIDIKVEQAEFSDLNLVAHANGGFSVDMEVLRNGVKVV-RSLKPDFVLIRQHAFSMARNGDYRSLVIGLQYAGIPSVNSLHSVYNFCDKPWVFAQMVRLHKKLGTEEFPLIDQTFYPNHKEM-----LSSTTYPV 266**

**HsSynIIa KVLLVVDEPHADWAKCFRGKKVL-GDYDIKVEQAEFSELNLVAHADGTYAVDMQVLRNGTKVV-RSFRPDFVLIRQHAFGMAENEDFRHLIIGMQYAGLPSINSLESIYNFCDKPWVFAQLVAIYKTLGGEKFPLIEQTYYPNHKEM-----LTLPTFPV 266**

**HsSynIIb KVLLVVDEPHADWAKCFRGKKVL-GDYDIKVEQAEFSELNLVAHADGTYAVDMQVLRNGTKVV-RSFRPDFVLIRQHAFGMAENEDFRHLIIGMQYAGLPSINSLESIYNFCDKPWVFAQLVAIYKTLGGEKFPLIEQTYYPNHKEM-----LTLPTFPV 266**

**HsSynIIIa RILLVIDDAHTDWSKYFHGKKVN-GEIEIRVEQAEFSELNLAAYVTGGCMVDMQVVRNGTKVVSRSFKPDFILVRQHAYSMALGEDYRSLVIGLQYGGLPAVNSLYSVYNFCSKPWVFSQLIKIFHSLGPEKFPLVEQTFFPNHKPM-----VTAPHFPV 245**

**HsSynIIIb RILLVIDDAHTDWSKYFHGKKVN-GEIEIRVEQAEFSELNLAAYVTGGCMVDMQVVRNGTKVVSRSFKPDFILVRQHAYSMALGEDYRSLVIGLQYGGLPAVNSLYSVYNFCSKPWVFSQLIKIFHSLGPEKFPLVEQTFFPNHKPM-----VTAPHFPV 245**

**HsSynIIIc RILLVIDDAHTDWSKYFHGKKVN-GEIEIRVEQAEFSELNLAAYVTGGCMVDMQVVRNGTKVVSRSFKPDFILVRQHAYSMALGEDYRSLVIGLQYGGLPAVNSLYSVYNFCSKPWVFSQLIKIFHSLGPEKFPLVEQTFFPNHKPM-----VTAPHFPV 245**

**8 C D E**

**NvSyn** **VVKVGHANSGYGKVCAQNHRTFQDIASIVA----LTDTYATTE--PFVAGKCDIRIQKIGDHLRAFKRT-SISDNWKTNTGSAVLEQIEITDRYKLWAEECSKLFGGLDIMCVEAILGKDKKEYIIEVTDTAMRLFNDTVDEDQQRIADLVIQKMEAHY 360**

**Cesnn1a VVSVNEGFQGIGKIKVNNHEELCDVEGMLQI---MSKGDTEVEVQPFVDAKYDLHIQKIGHEYKTFIRR-GICKHWKSNVGSSVLEQISTCERHKKYLKAITDHVGPMHICSIDILVSKEGREFVHDVND-VIAYFGESAEDDRRAASLLLRALVAPRI 302**

**Cesnn1b VVSVNEGFQGIGKIKVNNHEELCDVEGMLQI---MSKGDTEVEVQPFVDAKYDLHIQKIGHEYKTFIRR-GICKHWKSNVGSSVLEQISTCERHKKYLKAITDHVGPMHICSIDILVSKEGREFVHDVND-VIAYFGESAEDDRRAASLLLRALVAPRI 375**

**DmSyna VLKAGHCHGGVATARLENQSALQDAAGLVSGAGNDSHCYCTIE--PYIDAKFSVHIQKIGNNYKAFMRK-SITGNWKTNQGSAMLEQITLTEKYKSWVDEISELFGGMEVCGLSVVVAKDGREYIISACDSTFALIGDTQEEDRRQIADLVSGRM-QNV 395**

**DmSync VLKAGHCHGGVATARLENQSALQDAAGLVSGAGNDSHCYCTIE--PYIDAKFSVHIQKIGNNYKAFMRK-SITGNWKTNQGSAMLEQITLTEKYKSWVDEISELFGGMEVCGLSVVVAKDGREYIISACDSTFALIGDTQEEDRRQIADLVSGRM-QVE 395**

**DmSyne VLKAGHCHGGVATARLENQSALQDAAGLVSGAGNDSHCYCTIE--PYIDAKFSVHIQKIGNNYKAFMRK-SITGNWKTNQGSAMLEQITLTEKYKSWVDEISELFGGMEVCGLSVVVAKDGREYIISACDSTFALIGDTQEEDRRQIADLVSGRM-QNV 455**

**AcSyn11.1 VVKIGHAHSGLGKVRINNHYDFQDLAGVVA----VTQGYSTTE--PFIDAKYDLHIQKIGPSYKAFVRK-SISGNWKANTGSAMLEQIAMNERFKLWVDECSQLFGGLDVVAVEAIHGKDGREHIIEVNGSSMTLLGEAQEEDRRLIAELVLAKM-QAM 376**

**AcSyn2.1 VVKIGHAHSGLGKVRINNHYDFQDLAGVVA----VTQGYSTTE--PFIDAKYDLHIQKIGPSYKAFVRK-SISGNWKANTGSAMLEQIAMNERFKLWVDECSQLFGGLDVVAVEAIHGKDGREHIIEVNGSSMTLLGEAQEEDRRLIAELVLAKM-QAM 389**

**AcSyn8.2 VVKIGHAHSGLGKIKIDTVHGFQDMASIVA----VTSSYATTE--PFVDSKYDIHVQKIGTNYKAYLRK-SISGNWKANTGSAMLEQIAMNERFKLWVDECSQLFGGLDVVAVEAIHGKDGREHIIEVNGSSMTLLGEAQEEDRRLIAELVLAKM-QAM 394**

**AcSyn7.1 VVKIGHAHSGLGKVRINNHYDFQDLAGVVA----VTQGYSTTE--PFIDAKYDLHIQKIGPSYKAFVRK-SISGNWKANTGSAMLEQIAMNERFKLWVDECSQLFGGLDVVAVEAIHGKDGREHIIEVNGSSMTLLGEAQEEDRRLIAELVLAKM-QAM 381**

**LpSyn-long VVKIGHAHSGMGKVKVDHHHAFQDIASVVA----VTKSYATTE--PYIDCKCDIHVQKIGNNYKAFLRK-SISGNWKANTGSAMLEQIQMNEKYKLWVDECSQLFGGLDIVAVEALQGKDGREYIIEVNDSSMALLGETQEEDRRLIAEMVLQKM-HMY 394**

**LpSyn-short VVKIGHAHSGMGKVKVDHHHAFQDIASVVA----VTKSYATTE--PYIDCKCDIHVQKIGNNYKAFLRK-SISGNWKANTGSAMLEQIQMNEKYKLWVDECSQLFGGLDIVAVEALQGKDGREYIIEVNDSSMALLGETQEEDRRLIAEMVLQKM-HMY 394**

**HpSyn VVKIGHAHRGLGKIKVDNVQTLEDLASVMA----TMSSYATTE--PFIDSKYDIHVQKIGTNYKAYLRK-SIAGNWKANTGSAMLEQIPMDERFKLWADECSQLFGGLDVVSVEAIQGKDGRDHIIEVNGSSMALLGEAQEEDRRLISEMVMAKM-QMM 373**

**CcSyn VVKIGHAHSGMGKVKINNHYDFQDIASVVA----VTNCYSSTE--PFVDGKYDIHIQKIGSNYKAFMRK-SISGNWKANTGSAMLEQITMTERYKTWVDECSQLFGGLDMLAVEGIHGKDGKEYIIEVNDSSMGLLGESQEEDRRLIADLVLAKM-EVY 392**

**SpSyn VIKIGHAHAGMGKVKVDNHQDFQDIGSVIA----ITNCYATTE--SFLDAQYDIRVQKIGNTYKAFFRRTSISGNWKANTGSAVVEEMPMNGRFKLWIDEAAELFGGMDIVTVEAIHTKDGKDFIIEVNDSSMCLMGEKQEDDMKAIAELVIQKMHAVI 370**

**AmphiSyn-long VVKIGHAHAGLGKVKVENHHDFQDIASVVA----VANTYATTE--PFIDAKHDIRVQKIGNNYKAYMRT-SISGNWKANTGSAMLEQIPMTEKYRVWVDAVSEIFGGLDICAVEAIHGKDGKDYIIEVNDSTMPLLGENQEEDRQLISDVVLQRMTQ-V 374**

**AmphiSyn-short VVKIGHAHAGLGKVKVENHHDFQDIASVVA----VANTYATTE--PFIDAKHDIRVQKIGNNYKAYMRT-SISGNWKANTGSAMLEQIPMTEKYRVWVDAVSEIFGGLDICAVEAIHGKDGKDYIIEVNDSTMPLLGENQEEDRQLISDVVLQRMTQ-V 374**

**Ci-Syn VLKIGHAHRGMAKFKVENHYEFQDVVSVAA----LTNAYVVSE--TFIDSAYDIRVQKIGGNYKSYIRT-SISKNWKANTGSAMLEETPVTERHKLWVDACSEMFGGLDIVAVKAVHGKDGRDYIIEVVDCSMPLIGERQEEDRRMISDLVMQRMTA-C 403**

**HsSynIa VVKMGHAHSGMGKVKVDNQHDFQDIASVVA----LTKTYATAE--PFIDAKYDVRVQKIGQNYKAYMRT-SVSGNWKTNTGSAMLEQIAMSDRYKLWVDTCSEIFGGLDICAVEALHGKDGRDHIIEVVGSSMPLIGDHQDEDKQLIVELVVNKMAQAL 418**

**HsSynIb VVKMGHAHSGMGKVKVDNQHDFQDIASVVA----LTKTYATAE--PFIDAKYDVRVQKIGQNYKAYMRT-SVSGNWKTNTGSAMLEQIAMSDRYKLWVDTCSEIFGGLDICAVEALHGKDGRDHIIEVVGSSMPLIGDHQDEDKQLIVELVVNKMAQAL 418**

**HsSynIIa VVKIGHAHSGMGKVKVENHYDFQDIASVVA----LTQTYATAE--PFIDSKYDIRVQKIGNNYKAYMRT-SISGNWKTNTGSAMLEQIAMSDRYKLWVDTCSEMFGGLDICAVKAVHGKDGKDYIFEVMDCSMPLIGEHQVEDRQLITELVISKMNQLL 418**

**HsSynIIb VVKIGHAHSGMGKVKVENHYDFQDIASVVA----LTQTYATAE--PFIDSKYDIRVQKIGNNYKAYMRT-SISGNWKTNTGSAMLEQIAMSDRYKLWVDTCSEMFGGLDICAVKAVHGKDGKDYIFEVMDCSMPLIGEHQVEDRQLITELVISKMNQLL 418**

**HsSynIIIa VVKLGHAHAGMGKIKVENQLDFQDITSVVA----MAKTYATTE--AFIDSKYDIRIQKIGSNYKAYRRT-SISGNWKANTGSAMLEQVAMTERYRLWVDSCSEMFGGLDICAVKAVHSKDGRDYIIEVMDSSMPLIGEHVEEDRQLMADLVVSKMSQ-L 396**

**HsSynIIIb VVKLGHAHAGMGKIKVENQLDFQDITSVVA----MAKTYATTE--AFIDSKYDIRIQKIGSNYKAYRRT-SISGNWKANTGSAMLEQVAMTERYRLWVDSCSEMFGGLDICAVKAVHSKDGRDYIIEVMDSSMPLIGEHVEEDRQLMADLVVSKMSQ-L 396**

**HsSynIIIc VVKLGHAHAGMGKIKVENQLDFQDITSVVA----MAKTYATTE--AFIDSKYDIRIQKIGSNYKAYRRT-SISGNWKANTGSAMLEQVAMTERYRLWVDSCSEMFGGLDICAVKAVHSKDGRDYIIEVMDSSMPLIGEHVEEDRQLMADLVVSKMSQ-L 396**

6 7

DOMAIN D

**6 7 2**

**NvSyn**  **PPTIQATSPTKAALSGAF---------------------------------------------------------------------------------------------------------------------------------------------- 378**

**Cesnn1a TSPIA-AEPPATSPTTSA-------------------------------------------------------VTNGHGNHGPAPAVPTRRLPPHPSSSSAAP--------------------------------------------------------- 349**

**Cesnn1b TSPIA-AEPPATSPTTSA-------------------------------------------------------VTNGHGNHGPAPAVPTRRLPPHPSSSSAAP--------------------------------------------------------- 422**

**DmSyna CRPSM-AQTGPGKLPSRS-----------SVSSRAESPTDEGVAPTPPLPAGP-----RPAPMGGPPPIPERTSPAVGSIGRLSSRSSISEVPEEPSSSGPST--------------------------------------------------------- 481**

**DmSync RLPS-QHGADGSGQVAL---------------------PLLGLFPSRESHGRG-------------------RGSNTTTPSWTKTRAHGWTTTDTGAYLTRRG--------------------------------------------------------- 457**

**DmSyne CRPSM-AQTGPGKLPSRS-----------SVSSRAESPTDEGVAPTPPLPAGP-----RPAPMGGPPPIPERTSPAVGSIGRLSSRSSISEVPEEPSSSGPST--------------------------------------------------------- 541**

**AcSyn11.1 CKPVQ-TSMSKATSSGAI---------MHQVNGS-----HSGPQAGLRSSHAP------------------RKPGQGRGHDGGPPPQGPMRAPGMPGGPPAPVP-------------------------------------------------------- 447**

**AcSyn2.1 CKPVQ-TSMSKATSSGAI---------MHQVNGS-----HSGPQAGLRSSHAP------------------RKPGQGRGHDGGPPPQGPMRAPGMPGGPPAPVP-------------------------------------------------------- 460**

**AcSyn8.2 CKPVQ-TSMSKATSSGAI---------MHQVNGS-----HSGPQAGLRSSHAP------------------RKPGQGRGHDGGPPPQGPMRAPGMPGGPPAPVP-------------------------------------------------------- 465**

**AcSyn7.1 CKPVQ-TSMSKATSSGAI---------MHQVNGS-----HSGPQAGLRSSHAP------------------RKPGQGRGHDGGPPPQGPMRAPGMPGGPPAPVP-------------------------------------------------------- 452**

**LpSyn-long CKP---NTMSQAMSSGTI---------QSAADST-----ATPPPPPPRPASSR------------------PPPPP---ESGGPPSQPPPRAPGRQ-MPPGQGP-------------------------------------------------------- 459**

**LpSyn-short CKP---NTMS------------------------------------------------------------------------GPPSQPPPRAPGRQ-MPPGQGP-------------------------------------------------------- 422**

**HpSyn CKPAQ-QPLSKASSSQSI---------TPQANGA-----QKPVLAASPSRQAQ------------------GRPLDTSAQATPGQARGPPSSGGLPGVSNSQTPLSN----------------------------------------------------- 447**

**CcSyn CKPVQ-TTISKTTSAGAL-------MHYAMNGEP-----ERGLPDPSMAPPPPQAQQPPPPQMRGAPPQRQ-----MSTPTGAMGGGSPRSAPGPDRGPPQQRAG------------------------------------------------------- 479**

**SpSyn IKNT--IRPSNNHTPNSVR--------------------------------------------------------------------------------------------------------------------------------------------- 387**

**AmphiSyn-long CRAGA-SAAQNINPHNAW----------------------------------------------------RRVLVMSGLSTDAEPAEPQTSAKSMTGFLNLGG--------------------------------------------------------- 424**

**AmphiSyn-short CRAGA-SAAQNIN-------------------------------------------------------------------------------KSMTGFLNLGG--------------------------------------------------------- 397**

**Ci-Syn TRPR--PDHTPSTAASVY---------------------GRSYSSQNTSREMT-------------------PQRNVQIPQNRPNHAIPSCPPSAPLSSHSSE--------------------------------------------------------- 464**

**HsSynIa PRQRQ-RDASPGRGSHGQTPSPGALPLGRQTSQQPAGPPAQQRPPPQGGPPQP-----GPGPQRQGPPLQQRPPPQGQQHLSGLGPPAGSPLPQRLPSPTSAPQQPASQAAPPTQGQGRQSRPVAGGPGAPPAARPPASPSPQRQAGPPQATRQTSVSGP 572**

**HsSynIb PRQRQ-RDASPGRGSHGQTPSPGALPLGRQTSQQPAGPPAQQRPPPQGGPPQP-----GPGPQRQGPPLQQRPPPQGQQHLSGLGPPAGSPLPQRLPSPTSAPQQPASQAAPPTQGQGRQSRPVAGGPGAPPAARPPASPSPQRQAGPPQATRQTSVSGP 572**

**HsSynIIa SRT---PALSPQRPLTTQQPQSG-----------TLKDPDSSKTPPQR------------------------PPPQGG-----PGQPQGMQPPGKVLPPRRLP--------------------------PGPSLPPSSSSSSSSSSSAPQ---------- 499**

**HsSynIIb SRT---PALSPQRPLTTQQPQSG-----------TLKDPDSSKTPPQR------------------------PPPQG--------------CLQYILDCNGIA--------------------------VGPKQVQAS---------------------- 478**

**HsSynIIIa PMPGG-TAPSPLRPWAPQIK--------------SAKSPGQAQLGPQLGQPQP------------------RPPPQGG------PRQAQSPQPQRSGSPSQQRLSPQ---------------------GQQPLSPQSGSPQQQRSPGSPQLSRASSGSSP 496**

**HsSynIIIb PMPGG-TAPSPLRPWEALAKL-------------SLLSPRDLEAPPNRGSPHK------------------ASSP------------------------------------------------------------------------------------- 439**

**HsSynIIIc PMPGG-TAPSPLRPWAPQIK--------------SAKSPGQAQLGPQLGQPQP------------------RPPPQAN------LSPA------------------------------------------------------------------------ 445**

DOMAIN E

**3 X Y**

**NvSyn ---------------------------------------------------------------------------------SIAGSPGAPAASAAASD----------------KSNVDEKTKNGAFSGIL-D- 413**

**Cesnn1a -----------------------------------------------------------------------PRGHMSDKVESRHKDHYDPPPQIPRTGSRESVS----------YVDDTMGQLKRTFAGFFGE- 401**

**Cesnn1b -----------------------------------------------------------------------PRGHMSDKVESRHKDHYDPPPQIPRTGSRESVS----------YVDDTMGQLKRTFAGFFGE- 474**

**DmSyna -----------------------------------------------------------------------VGGVRRDSQTSQSSTISSSVSRAGQRPPQTQNSVV-------EDAEDTMKNLRKTFAGIFGDM 537**

**DmSync -----------------------------------------------------------------------FHWAAEQSQQHFGGAGGTLLVGTQHSGWGAS-------------------------------- 488**

**DmSyne -----------------------------------------------------------------------VGGVRRDSQTSQSSTISSSVSRAGQRPPQTQNSVV-------EDAEDTMKNLRKTFAGIFGDM 597**

**AcSyn11.1 -----------------------------------------------------------------------RPRHMNNPPPQPFPGQGR-PQ-GCSACASK-------------DEEDTMKNLRKTFAGIFGDM 495**

**AcSyn2.1 -----------------------------------------------------------------------RPRHMNNPPPQPFPGQGR-PQ-GCSACASK-------------DEEDTMKNLRKTFAGIFGDM 508**

**AcSyn8.2 -----------------------------------------------------------------------RPRHMNNPPPQPFPGQGR-PQ-GCSACASK-------------DEEDTMKNLRKTFAGIFGDM 513**

**AcSyn7.1 -----------------------------------------------------------------------RPRHMNNPPPQPFPGQGR-PQ-GCSACASK-------------DEEDTMKNLRKTFAGIFGDM 500**

**LpSyn-long -----------------------------------------------------------------------PPGHPSQVPP----GQGP-PQPMTSMGQS--------------QDEDTMQNLRKTFAGIFGDV 503**

**LpSyn-short -----------------------------------------------------------------------PPGHPSQVPP----GQGP-PQPMTSMGQS--------------QDEDTMQNLRKTFAGIFGDV 466**

**HpSyn -----------------------------------------------------------------------QPSHLSNPPPQPFPTSTSGPQ-GLPRMASK-------------DEEDTMKNLRKTFAGIFGDV 496**

**CcSyn -----------------------------------------------------------------------PPGQRPNAPDSLPRGQAPPPMPRPQGPASPGTVEAIARELQEPDQEDTMKNLRKTFAGIFGDM 542**

**SpSyn -----------------------------------------------------------------------SLGAASTTSTSNATAGGSQASSSARATP--------------TEDEDTFKNLKKTFASIFGDL 436**

**AmphiSyn-long -----------------------------------------------------------------------SASAPPSASSSHSTLPGAQAGAAGPPPA-------------DEDQAESIRKLRKAFSGIFGEP 474**

**AmphiSyn-short -----------------------------------------------------------------------SASAPPSASSSHSTLPGAQAGAAGPPPA-------------DEDQAESIRKLRKAFSGIFGEP 447**

**Ci-Syn -----------------------------------------------------------------------PPSPTATRRSNSVSEPSPPGGDRERIRSTTVSE-------DKESKAEKIRSLRQSFANLFSD- 519**

**HsSynIa APPKASGAPPGGQQRQGPPQKPPGPAGPTRQASQAGPVPRTGPPTTQQPRPSGPGPAGRPKPQLAQKPSQDVPPPATAAAGGPPHPQLNKSQSLTNAFNLPEPAPPRPSLSQDEVKAETIRSLRKSFASLFSD- 705**

**HsSynIb APPKASGAPPGGQQRQGPPQKPPGPAGPTRQASQAGPVPRTGPPTTQQPRPSGPGPAGRPKPQLAQKPSQDVPPPATAAAGGPPHPQL-KASPAQAQP------------------------------------ 669**

**HsSynIIa ---------------------------------------RPGGPTTHGDAPSSSSSLAEAQPPLAAPPQ-----------KPQPHPQLNKSQSLTNAFSFSESSFFRSSANEDEAKAETIRSLRKSFASLFSD- 582**

**HsSynIIb -------------------------------------------------------------------------------------------------------------------------------------- 478**

**HsSynIIIa NQ---------------------ASKPGATLASQPRP-PVQGRSTSQQGEESKK-PA-------------------------PPHPHLNKSQSLT-NSLSTSDTSQRGTPSEDEAKAETIRNLRKSFASLFSD- 580**

**HsSynIIIb -------------------------------------------------------------------------------------------------------------------------------------- 439**

**HsSynIIIc -------------------------------------------------------------------------------------------------------------------------------------- 445**
