# Supplementary material for: The synapsin gene family in basal chordates: evolutionary perspectives in metazoans
Source: BMC Evol Biol. 2010 Jan 29;10:32. doi: 10.1186/1471-2148-10-32 (PMC2825198; doi:10.1186/1471-2148-10-32)
Supplement: Additional file 4 — Sequence identity matrix of synapsin domains. Comparisons of domains A, C and E from synapsins of various species after alignment using GeneWorks (Clustal W method with PAM250 weighting and identical gap costs). Colored boxes indicate percent identity of A domain (in red), C domain (in green) and E domain (in yellow). [file 1471-2148-10-32-S4.PDF]

PERCENT IDENTITY

AcSyn			HpSyn			CcSyn			LpSyn			DmSyn			Cesnn1			NvSyn			SpSyn			AmphiSyn			Ci-Syn			HsSynI			HsSynII			HsSynIII			
			75.9	77.5	75.0	55.2	81.0	53.6	51.7	78.7	55.4	44.8	54.9	46.4	31.0	34.3	25.0	3.4	63.5	19.6	34.5	65.7	35.7	37.9	70.5	30.4	24.1	63.2	14.3	27.6	65.7	26.8	24.1	67.6	25.0	24.1	64.8	25.0	**AcSyn**
						55.2	68.9	48.2	55.2	70.2	50.0	41.4	51.4	41.1	27.6	31.1	23.2	3.4	54.3	21.4	27.6	60.0	37.5	31.0	62.2	32.1	27.6	61.6	14.3	24.1	61.3	26.8	27.6	61.6	26.8	27.6	59.0	25.0	**HpSyn**
									55.2	79.4	55.4	44.8	56.2	44.6	34.5	33.3	26.8	3.4	65.7	19.6	34.5	62.2	35.7	31.0	72.4	28.6	31.0	63.2	21.4	31.0	62.9	26.8	34.5	67.3	25.0	34.5	65.1	25.0	**CcSyn**
												51.7	55.9	46.4	31.0	31.7	30.4	10.3	61.9	19.6	27.6	65.4	33.9	24.1	71.4	32.1	27.6	61.9	16.1	17.2	64.8	33.9	20.7	67.6	23.2	20.7	64.1	26.8	**LpSyn**
															31.0	29.5	26.8	3.4	48.3	19.6	24.1	46.3	41.1	24.1	54.0	33.9	34.5	48.9	19.6	20.7	50.5	32.1	24.1	50.5	33.9	24.1	50.2	41.1	**DmSyn**
																		3.4	27.9	14.3	20.7	31.4	23.2	24.1	30.8	23.2	24.1	31.4	16.1	17.2	30.8	21.4	20.7	30.5	17.9	20.7	30.2	19.6	**Cesnn1**
																					6.9	57.8	17.9	13.8	63.2	25.0	6.9	54.9	10.7	6.9	57.1	12.5	6.9	61.0	16.1	6.9	57.8	16.1	**NvSyn**
																								58.6	64.1	26.8	44.8	61.6	14.3	51.7	61.6	28.6	44.8	62.2	28.6	44.8	63.5	28.6	**SpSyn**
																											58.6	62.2	23.2	62.1	65.1	39.3	55.2	68.6	35.7	58.6	67.6	35.7	**AmphiSyn**
																														51.7	62.5	35.7	51.7	66.3	35.7	51.7	66.3	32.1	**Ci-Syn**
																																	86.2	78.4	73.2	89.7	71.1	66.1	**HsSynI**
																																				93.1	73.0	64.3	**HsSynII**
																																							**HsSynIII**
