# Supplementary material for: The synapsin gene family in basal chordates: evolutionary perspectives in metazoans
Source: BMC Evol Biol. 2010 Jan 29;10:32. doi: 10.1186/1471-2148-10-32 (PMC2825198; doi:10.1186/1471-2148-10-32)
Supplement: Additional file 5 — Other synapsin sequences used in this study. Identification of synapsin-related sequences in two cnidarians (Hydra magnipapillata and Acropora millepora) and two protozoans (Naegleria gruberi and Entamoeba histolytica). [file 1471-2148-10-32-S5.PDF]

Species name (Taxonomy)	Genome database used	Accession or data reference
Hydra magnipapillata (Cnidaria)	Traces NCBI, GenBank, Hydra EST database	Not determined
Acropora millepora (Cnidaria)	Trace NCBI	Not determined
Trichoplax adhaerens (Placozoa)	Trace NCBI	Not found
Monosiga brevicollis (Choanoflagellates)	Traces NCBI, JGI	Not found
Amphimedon queenslandica (Porifera)	Traces NCBI	Not found
Naegleria gruberi (Excavates, Heterolobosea)	JGI	scaffold_19:385514-386710
Entamoeba histolytica (Amoebozoa)	GenBank	XP_652171
