# Supplementary material for: The synapsin gene family in basal chordates: evolutionary perspectives in metazoans
Source: BMC Evol Biol. 2010 Jan 29;10:32. doi: 10.1186/1471-2148-10-32 (PMC2825198; doi:10.1186/1471-2148-10-32)
Supplement: Additional file 6 — Multiple alignment of Nematostella, amphioxus, Ciona, human and protozoa synapsins. This file contains a multiple alignment of C-domain of amphioxus, Ciona, human synapsin and synapsin full-length sequences of Naegleria gruberi and Entamoeba histolytica. [file 1471-2148-10-32-S6.PDF]

DOMAIN C

```
EntamoebaSyn       MSINKVKKLLVIGGRPTINWYKLFKN-TSIG-NYSIEVEFTMWDEMVLTSYSDSGCIISIMPSKYAIPDTPMNTRRTFQPDFLLIRGACQGVYG-QDWKNELLGLMYCGIPSINSLESLY 117
NaegleriaSyn       -----MSSIIDYNQSDQSNWYKLFANKTTKQ-GKKIKVEQAGWEEIKLVSNSEYGPILELKAKNDSMFSQ-QSKDRDFKPDFVLVRNFPSSLHT-ETFRNVLLGLMFANIPAVNSLESVY 112
NematostellaSyn    -------KTLLIIDSADHDWSKIFKGRNLHNGSYGIRVEQAEFADINLASYSDSGTMVDIQVFR----EGTVVV-RSFRPDFVLVRQSIRGIGPREDYRHILLGLQFGNVPSVNSLESIY 162
AmphioxusSyn-long  -------KTLLVIDDQHTDWGKYFRGKRINN-EYEIRVEQADFSEINLAAYSDTGTMIDMQINR----QGTKVV-RSFRPDFVLVRQHCRGLDANQDYRSVILGLRWGAIPSVNTLLSIY 177
CionaSyn           -------KTVLVVDEQHTDWSKYLKGRKIHN-DYTVRVEQAEFSELSLSSHSVNGVTVDINCYR----NGNKVV-RSFKPDFLLVRQTPRSMAQGEDFRNLVIGLKYGGIPSVNSLHSQF 206
HumanSynIa         -------RVLLVIDEPHTDWAKYFKGKKIHG-EIDIKVEQAEFSDLNLVAHANGGFSVDMEVLR----NGVKVV-RSLKPDFVLIRQHAFSMARNGDYRSLVIGLQYAGIPSVNSLHSVY 220
HumanSynIIa        -------KVLLVVDEPHADWAKCFRGKKVLG-DYDIKVEQAEFSELNLVAHADGTYAVDMQVLR----NGTKVV-RSFRPDFVLIRQHAFGMAENEDFRHLIIGMQYAGLPSINSLESIY 220
HumanSynIIIa       -------RILLVIDDAHTDWSKYFHGKKVNG-EIEIRVEQAEFSELNLAAYVTGGCMVDMQVVR----NGTKVVSRSFKPDFILVRQHAYSMALGEDYRSLVIGLQYGGLPAVNSLYSVY 199

EntamoebaSyn       MCLEKPVIYSKLLQIHKQYK--EKFPLIPQTYYPSWSSMSFN--------TGFPLVAKVGTVHAGFGKMKLENQEDFDDLVSLIALQNRYVTTEPYIKWDYDFRIQKIGDHYRAFQRVS- 226
NaegleriaSyn       MCSEKPIIYGKLKEIQKKLGGFENFPLIPQIYYPNIRSTDFFDENEKTTPQEFPVVCKVGTVHAGFGKQKLDTKSSFTDFTTVLALYKDYFTCEPFCQVDYDLRLQKIGNHYRVYQRSSD 232
NematostellaSyn    NFAEKPWVFSQLIQIKKRLGK-EEFPLIEQAYYPNHKEMLIT--------PRFPVVVKVGHANSGYGKVCAQNHRTFQDIASIVALTDTYATTEPFVAGKCDIRIQKIGDHLRAFKRTSI 273
AmphioxusSyn-long  NFMEKPWVYAHLLQIRKRIGK-EKFPLIDRAYYPNHKEMLIT--------PKFPVVVKIGHAHAGLGKVKVENHHDFQDIASVVAVANTYATTEPFIDAKHDIRVQKIGNNYKAYMRTSI 288
CionaSyn           NFMDKPWTFSQLIRIQKRLGP-EKFPLVEQTYYPSHKQMLTS--------SHFPVVLKIGHAHRGMAKFKVENHYEFQDVVSVAALTNAYVVSETFIDSAYDIRVQKIGGNYKSYIRTSI 317
HumanSynIa         NFCDKPWVFAQMVRLHKKLGT-EEFPLIDQTFYPNHKEMLSS--------TTYPVVVKMGHAHSGMGKVKVDNQHDFQDIASVVALTKTYATAEPFIDAKYDVRVQKIGQNYKAYMRTSV 331
HumanSynIIa        NFCDKPWVFAQLVAIYKTLGG-EKFPLIEQTYYPNHKEMLTL--------PTFPVVVKIGHAHSGMGKVKVENHYDFQDIASVVALTQTYATAEPFIDSKYDIRVQKIGNNYKAYMRTSI 331
HumanSynIIIa       NFCSKPWVFSQLIKIFHSLGP-EKFPLVEQTFFPNHKPMVTA--------PHFPVVVKLGHAHAGMGKIKVENQLDFQDITSVVAMAKTYATTEAFIDSKYDIRIQKIGSNYKAYRRTSI 310

EntamoebaSyn       -SCWKG--KGMNQTDNDVPVTDEYKRYIDLASKALG-MDICALDGIHDPITNKNYIIELNDSAIGLVQRHVEEDLNYIKELVLSRIKQIYKL 314
NaegleriaSyn       -SSWKNNWGAMKFKALDT-IEERYKLWMDEVSQLFGGLDLFTLD-VMKLKDGSERIIELNDSSMGLLYEYETQDNLCIVETVMDKIHSLD-L 320
NematostellaSyn    SDNWKTN--TGSAVLEQIEITDRYKLWAEECSKLFGGLDIMCVEAILGK-DKKEYIIEVTDTAMRLFNDTVDEDQQRIADLVIQKMEAHYPP 362
AmphioxusSyn-long  SGNWKAN--TGSAMLEQIPMTEKYRVWVDAVSEIFGGLDICAVEAIHGK-DGKDYIIEVNDSTMPLLGENQEEDRQLISDVVLQRMTQVCRA 377
CionaSyn           SKNWKAN--TGSAMLEETPVTERHKLWVDACSEMFGGLDIVAVKAVHGK-DGRDYIIEVVDCSMPLIGERQEEDRRMISDLVMQRMTACTRP 406
HumanSynIa         SGNWKTN--TGSAMLEQIAMSDRYKLWVDTCSEIFGGLDICAVEALHGK-DGRDHIIEVVGSSMPLIGDHQDEDKQLIVELVVNKMAQALPR 420
HumanSynIIa        SGNWKTN--TGSAMLEQIAMSDRYKLWVDTCSEMFGGLDICAVKAVHGK-DGKDYIFEVMDCSMPLIGEHQVEDRQLITELVISKMNQLLSR 420
HumanSynIIIa       SGNWKAN--TGSAMLEQVAMTERYRLWVDSCSEMFGGLDICAVKAVHSK-DGRDYIIEVMDSSMPLIGEHVEEDRQLMADLVVSKMSQLPMP 399
```
